# Dispersive transport dynamics in porous media emerge from local correlations

**DOI:** 10.1038/s41467-022-33485-5

**Published:** 2022-10-06

**Authors:** Felix J. Meigel, Thomas Darwent, Leonie Bastin, Lucas Goehring, Karen Alim

**Affiliations:** 1 Max Planck Institute for Dynamics and Self-Organisation, Göttingen, DE-37077 Germany; 2 Max Planck Institute for the Physics of Complex Systems, Dresden, DE-01087 Germany; 3 School of Science and Technology, Nottingham Trent University, Nottingham, NG11 8NS UK; 4 Center for Protein Assemblies (CPA), Physik-Department, Technische Universität München, Garching b. München, DE-85748 Germany

**Keywords:** Statistical physics, Fluid dynamics, Permeation and transport

## Abstract

Understanding and controlling transport through complex media is central for a plethora of processes ranging from technical to biological applications. Yet, the effect of micro-scale manipulations on macroscopic transport dynamics still poses conceptual conundrums. Here, we demonstrate the predictive power of a conceptual shift in describing complex media by local micro-scale correlations instead of an assembly of uncorrelated minimal units. Specifically, we show that the non-linear dependency between microscopic morphological properties and macroscopic transport characteristics in porous media is captured by transport statistics on the level of pore junctions instead of single pores. Probing experimentally and numerically transport through two-dimensional porous media while gradually increasing flow heterogeneity, we find a non-monotonic change in transport efficiency. Using analytic arguments, we built physical intuition on how this non-monotonic dependency emerges from junction statistics. The shift in paradigm presented here broadly affects our understanding of transport within the diversity of complex media.

## Abstract (web summary)

Dispersive transport through complex media, relevant for semiconductors, liquid crystals, and biological soft matter, is influenced by their microscopic, porous structure. The authors consider the statistics of pore-junction units, in contrast to individual pores, to link morphology and macroscopic transport characteristics.

## Introduction

Dispersive transport through complex media has been in the focus of researchers across disciplines, with applications ranging from semiconductors^1–3^, to liquid crystals^4,5^, and biological soft matter^6,7^. Among those applications, fluid flow driven transport through porous media emerged as a paradigmatic system for the study of dispersive transport dynamics^8–11^: beside its experimental tractability, transport through porous media by the means of fluid flow is omnipresent in both inanimate and living systems, whenever manipulating, controlling, and retracing transport are central concerns. On the technical side, applications range from oil recovery to carbon geosequestration and chemical reactors^12–19^, while on the biological side understanding the functioning of living systems, like vascular systems^20–24^, algal colonies, or biofilms^7,25^, crucially depends on tracing how resources spread through biological systems. Porous media show a broad range of different morphologies and modifying their pore network structure, either by internal or external influence, allows for altering and controlling transport^26,27^. Yet, how manipulations of porous media morphology on the micro-scale affect macroscopic transport characteristics is still hardly understood. What are the general mechanisms and control parameter that dictate dispersive transport dynamics and allow for efficient transport in porous media?

Porous media pose heterogeneous environments through which particles are transported by both advection and diffusion. Already in single pores, the interplay between these two transport modes gives rise to intricate transport dynamics, as prominently displayed by Taylor dispersion^28–32^, which enhances mixing rates. Transport through a pore network adds another level of complexity, as a vast heterogeneity of flow rates is a defining characteristic of porous media, and pores with drastically different flow rates frequently adjoin each other^33–35^. Though detailed physical insight on transport at the single pore scale is available, understanding transport on the macroscopic scale by scaling up and extending the methods used on the pore scale is inhibited by complexity and numerical costs^36–39^. To overcome this conceptual barrier, a variety of models have been developed to describe the permeability, flow, and transport through porous media directly on macroscopic levels^40–42^, including effective advection–diffusion equations^43^, fractional advection–diffusion equations^44^, continuous time random walk models^8,9,11,45–49^, and memory kernel approaches^50,51^. While these models have proved successful, especially in geological applications, they remain partly based on phenomenological model assumptions like empirically derived waiting time distributions or memory kernel choices. Yet, connecting changes in the pore statistics to macroscopic transport dynamics is critical for applications where a key challenge is to control transport by manipulating the microscopic structure^26,52^, including technical applications^16,17^ and the understanding of transport in biological systems^27^. While Darcy’s law^53^ estimates mean transport dynamics, here often the central task is to optimise the efficiency of solute transport. Increasing the efficiency implies minimising the dispersive spread and thus deviations from the mean transport dynamics^54,55^: decreased variability in the transport increases the information quality of the mean transport dynamics, see also supplemental theory section 1.1. As porous media are often used in chemical reaction chambers to maximize the catalyst-fluid interface, a precise control of the dispersive spread is crucial to optimise chemical reaction cells^56–59^ and bio-technological applications^60,61^.

Investigating how manipulating the microscopic structure of porous media optimises transport efficiency, we uncover a change in paradigm: instead of using the statistics of individual pores, the statistics of pore junction units allows understanding of how macroscopic transport statistics emerge and provides information about both mean transport dynamics and the efficiency of transport. Analysing transport through pore junction units accounts for connections between pores with vastly different flow rates—details that are neglected by considering the statistics of individual pores. Combining experiments, simulations, and theory on two-dimensional porous media, we find that transport efficiency shows a counter-intuitive non-monotonic dependency on the heterogeneity of the pore space. Starting from a regular pore space, the transport efficiency initially increases, if the heterogeneity of the pore space is increased, until the efficiency ultimately decreases for large heterogeneity. Resolving this conundrum using the statistics of pore junctions, we offer broad insight into the physics of flow-based transport in porous media.

## Results

### Non-linear relation between dispersion and flow heterogeneity

To investigate how structural properties of porous media morphology determine the efficiency of transport, we perform microfluidic experiments and numerical simulations. To this end, we focus on two-dimensional model porous media where we modify the pore space architecture in a controlled manner. Reduction to two-dimensional media allows us to more precisely track the dispersion of fluorescent solutes in experiments and demonstrate the effects of heterogeneity. To manipulate the flow rate distribution, we vary the partitioning of flows at pore junctions by altering the porous medium disorder^35^. Circular obstacles are placed on a hexagonal lattice and are randomly perturbed in their positions by up to a fraction *χ* of the obstacle radius: the smaller the disorder parameter *χ*, the closer the porous medium is to a perfect lattice.

**Fig. 1:**
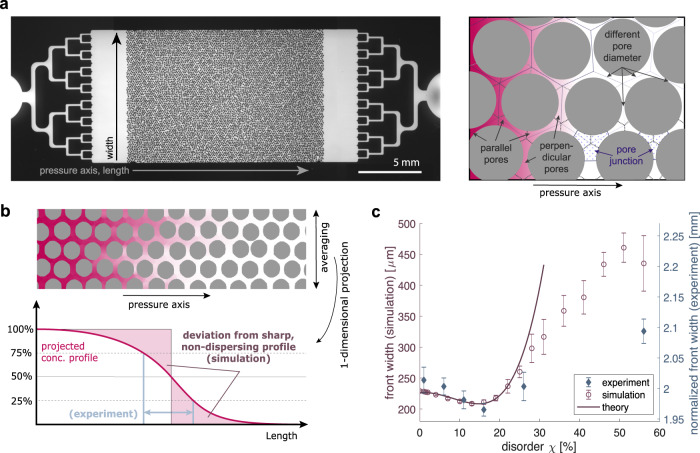
Pore-space heterogeneity affects transport dynamics. **a** Experimental setup. A two-dimensional porous medium is constructed inside a microfluidic chip. The effect of pore space heterogeneity on transport dynamics is investigated as a fluorescent solute traverses the chip. The inlet design is optimised to create initially sharp fronts. Parallel pores, perpendicular pores, and pore junctions are depicted in the cartoon to the right. **b** Measurement of the dispersive front width. Cartoon explaining the front width measures employed, see also ‘Methods’. The concentration profiles are averaged perpendicular to the pressure axis. The deviation from a step function measures the efficiency of transport. The distance between relative solute concentrations of 25% and 75% is the experimental measure for the front width. Each experimental run is corrected for an offset caused by diffusive spreading in the tubing. The average offset is added as a constant to match with the order of magnitude of visually obtained front widths in microscopy images. **c** Change of the dispersive front width with disorder in simulation and experiment. The dispersive front width is measured as a function of disorder in experiments (rhomb) and simulation (circle) for Pe_∥_ ≈ 30. Error bars show the standard deviations computed over a sample size of *n* = 6 for the simulation and *n* = 6 for the experimental data points *χ* = 1%, 11%, 16% and *n* = 5 for *χ* = 6%, 26%, 56%. The front width is evaluated when a relative solute concentration of 50% reaches the middle of the porous medium. The analytically derived functional form of Eq. (3) captures the non-monotonic dependency of the front width up to a disorder of *χ* ≈ 20%, as demonstrated by a fit over the simulation data points in the range *χ* ∈ [1%, 20%] with *R*^2^ = 0.97, see also supplemental theory sections 1.7 and 1.8. The non-monotonicity can be explained using pore junction statistics, but not by pore space statistics.

A series of microfluidic chips with different levels of disorder is created using templates of numerically generated obstacle positions; the same designs are later used for flow simulations. We optimised the chip inlets to generate a sharp front of fluorescent solute perpendicular to the applied pressure gradient in an area before the flow enters the porous medium, see Fig. 1a and ‘Methods’. The initially sharp solute front disperses as it propagates through the porous medium. The observable dispersion patterns differ depending on the chip’s disorder. To capture the differences in transport dynamics, we quantify the dispersion when the solute front reaches the middle of the chip by evaluating the concentration profile averaged perpendicular to the axis of the applied pressure gradient. To estimate the efficiency of transport, we quantify the dispersion by measuring the width of the averaged concentration profile. We define the front width as the distance between 25% and 75% of the full saturation level, see Fig. 1b. Strikingly, the efficiency of transport measured by the front width is a non-monotonic function of the disorder, see Fig. 1c. The front width surprisingly first *decreases* when introducing disorder among the obstacles. The averaged concentration profile is sharpest at intermediate disorder and only increases at high disorder, despite the roughness of concentration isolines monotonically increasing with disorder. This indicates that the most efficient transport is achieved at intermediate disorders and not at minimal disorder, as could be intuitively expected.

**Fig. 2:**
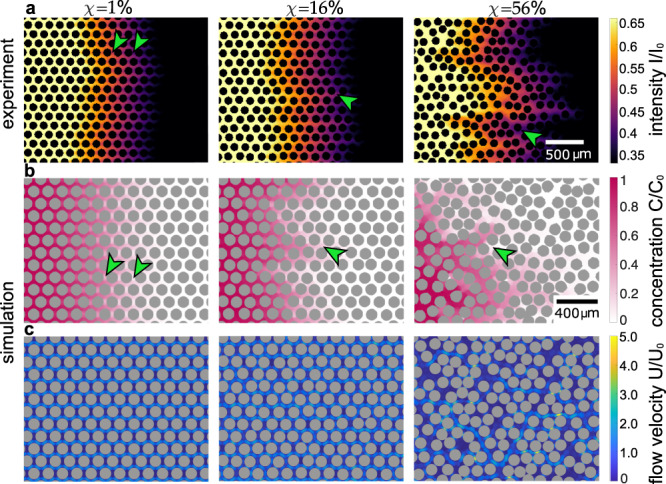
Dispersion dynamics differ qualitatively with disorder. **a** Visualisation of transport dynamics in experiments. The transport dynamics of a fluorescent solute are visualised for porous media with varying pore-space heterogeneity. Distinct transport characteristics emerge for different degrees of pore-space heterogeneity for a Péclet number of Pe_∥_ ≈ 30. Arrowheads highlight analogous dispersion characteristics between experiment and simulation. For *χ* = 1%, these highlight the slower fill-up of the perpendicular pores, compared to adjacent parallel pores. For *χ* = 16% the arrowheads point out the varying transport dynamics between adjacent lanes of parallel pores, and at *χ* = 56% they indicate extended regions of slower filling. **b** Visualisation of transport dynamics in simulations. The transport dynamics are contrasted to numerical simulations. Distinct transport characteristics for different degrees of pore-space heterogeneity are qualitatively recovered for a Péclet number of Pe_∥_ ≈ 30 and highlighted with arrowheads. **c** Visualisation of flow profiles in simulations. For the simulation, the transport dynamics and the underlying flow profiles are compared. With increasing disorder, the heterogeneity in the flow profiles is increased. The normalisation *U*_0_ denotes the average flow velocity in parallel pores, measured for a disorder of *χ* = 0%.

To corroborate our counter-intuitive experimental findings, we perform numerical simulations of solute dispersion and recover the experimentally observed non-monotonic behaviour of the front width as a function of porous media disorder, see Fig. 1c. The difference between simulation and experiment for *χ* = 56% stems from imprecision in the fabrication process of the chip for very narrow pores, where obstacles are placed closer than a distance *d* ≪ 10 μm. To gain intuition of how disorder affects dispersion, we compare front profiles obtained from simulations to front profiles obtained from microscopy images, see Fig. 2a, b and ‘Methods’. While we find clearly different dispersion patterns for different degrees of disorder, that qualitatively agree between experimental and simulation results, it is unclear how these patterns emerge and what their relevance for transport efficiency is. Here, simulations allow us to connect dispersion dynamics with the underlying flow heterogeneity. For this, we zoom into the dispersion dynamics on the pore level and distinguish pores with orientations parallel and perpendicular to the pressure gradient. We find qualitatively different dispersion dynamics for parallel and perpendicular pores, respectively, that correlate with higher or lower flow of the underlying flow field, as visualised by simulations, see Fig. 2b, c. Yet, how changes in the flow field relate to a non-monotonic change in front width and transport efficiency cannot be seen readily.

**Fig. 3:**
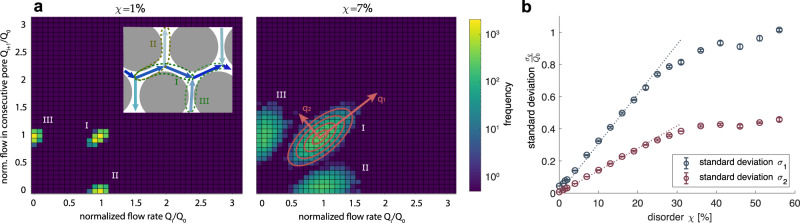
Flow statistics of porous media up to intermediate disorder are captured by Gaussian statistics. **a** Flow statistics of consecutive pores. The statistics of flow in consecutive pores for low disorder (*χ* = 1%) and intermediate disorder (*χ* = 7%) are plotted, defining the statistics of flow splitting at junctions. Flow statistics for porous media with a high degree of disorder are fundamentally different, see Supplementary Fig. S1. *Q*_0_ denotes the average flow rate in parallel high-flow pores evaluated at *χ* = 0%. For low and intermediate degrees of disorder three clusters are identified. Cluster *I*, containing information about the flow in consecutive parallel high-flow pores, gives the full information about the flow statistics at junctions. Computing the covariance matrix, we find that cluster *I* is well approximated by multivariate Gaussian statistics. The eigenvectors *q*_1_, *q*_2_ are independent of the degree of disorder (red arrows). The error ellipsoids in red are drawn at 0.5*σ*, 1*σ*, 1.5*σ* and 2*σ*. The flow statistics are well-captured by Gaussian statistics up to a disorder of 20%, see Supplementary Fig. S2. **b** Effect of disorder on flow statistics. The eigenvalues of the cluster *I* are plotted against the disorder of the porous media. Error bars show the standard deviation taken over 5 virtual replica. Both *σ*_1_ and *σ*_2_ increase initially linearly with disorder *χ* (dotted line). A linear regression was performed over the simulation data points in the range *χ* ∈ [2%, 22%] yielding a goodness of fit of *R*^2^ = 0.99 with slope *m*_1_ = 3.1 and *m*_2_ = 1.4 for *σ*_1_ and *σ*_2_, respectively. An analogous analysis showing a linear increase in the standard deviation of the flow rates obtained from experiments by particle image velocimetry is shown in Supplementary Fig. S7.

To connect flow field heterogeneity with transport efficiency, we map out how the partitioning of flows into parallel versus perpendicular pores changes as a function of porous medium disorder. Measuring the flow statistics of every pore with all its possible consecutive pores, we find different flow statistics depending on whether we look at intermediate or highly disordered porous media, see Fig. 3a and Supplementary Fig. S1. While the flow statistics of highly disordered porous media have been investigated in depth^33–35^, we focus here on porous media of low and intermediate disorder. Recall that we find the non-monotonic dependency between dispersion front width and disorder precisely in the range of low and intermediate disorder. In Fig. 3a, we observe three distinct clusters of junction flow statistics emerging for porous media of low and intermediate disorder. For our two-dimensional porous media, all pore junctions only connect three pores each. As a result, for low and intermediate disorder, pore junctions are perfectly defined as two consecutive parallel high-flow pores joining with a perpendicular low-flow pore. In Fig. 3a, the central cluster along the diagonal *I* represents two consecutive parallel (high flow) pores, while the off-diagonal clusters *II* and *III* represent a parallel pore feeding into a consecutive perpendicular (low flow) pore or vice versa.

As the flow in a perpendicular pore follows from the flows in the two parallel pores by fluid volume conservation, the central cluster *I* comprises all information about the flow statistics of junctions. To quantify how changes in junction flow statistics impact the non-monotonic front dispersion dynamics at low and intermediate disorder, we analyse the central cluster along the diagonal as a multivariate Gaussian distribution valid up to a disorder of ~20%, see Supplementary Fig. S2. The cluster’s covariance matrix eigenvectors are independent of the degree of disorder, and therefore the square root of the covariance matrix eigenvalues *σ*_1,2_ are representative of the standard deviation in flow statistics around the constant average flow *Q*_0_, see Supplementary Fig. S1. Importantly, the standard deviations *σ*_1,2_ both show monotonic behaviour, growing linearly with disorder *χ* up to a disorder of 20%, see Fig. 3b. We verify the linear increase in the standard deviation of the flow rates with increasing disorder in experiments by particle image velocimetry, see Supplementary Fig. S7. Note that the pore statistics can be extracted as the marginal distribution from the junction statistics. As such, the flow heterogeneity in the pore space is also a monotonically increasing function of heterogeneity. Consequently, we find that the flow statistics alone are insufficient to conclude on the efficiency of transport. With both the pore and the junction flow statistics monotonically increasing with disorder, the non-monotonic behaviour of the front width as a function of disorder cannot be understood intuitively. To make sense of the non-monotonicity, we need to make a conceptual shift to understand transport in terms of pore junction units, instead of individual pores, as the fundamental building block of porous media.

### Pore junctions as the minimal unit describe transport

To date, analytical theories of transport in porous media are based on transit time statistics through individual pores, as pioneered by Saffman^8,9^. In this approach, the statistics of how long particles spend time in individual pores is combined with the statistics of flow rates in the pore space to conclude on the transport dynamics through the full porous medium^11,45,47,49^. Yet these approaches neglect the coupling between jump direction and transit time contributions caused by back diffusion in pore bodies. Demonstrating that back diffusion and the direct interaction between low and high-flow pores are crucial to understanding transport efficiency, we perform a conceptual shift and derive transit time statistics for pore junctions as the minimal unit for transport dynamics through porous media. Specifically, we demonstrate that physical intuition can be generated by focusing directly on approximations of moments of the transit time instead of first deriving approximations for the transit time distribution and then recovering moment approximations.

Here, we tile the entire porous medium by our minimal unit, a junction block, which consists of two consecutive parallel pores denoted {∥, in} and {∥, out}, respectively, joined with a perpendicular pore {⊥}. By defining pore junctions, we combine the advantages of pore space descriptions and pore bodies. We accurately capture the interplay between pores with different flow rates in pore bodies.

The agreement of our numerical simulations with our experimental data in Fig. 2 already confirms that transport dynamics are successfully captured by Taylor dispersion in channel-like pores of varying diameter connected to a planar network. Analytically, transport through an individual pore *i* ∈ {∥, in; ∥, out; ⊥} is given by an advection–diffusion-equation:

1 $$\frac{\partial {c}_{i}(x,\;t)}{\partial t}=-{v}_{{{{{{{{\rm{eff}}}}}}}},i}(x)\frac{\partial {c}_{i}(x,\;t)}{\partial x}+{D}_{{{{{{{{\rm{eff}}}}}}}},i}(x)\frac{{\partial }^{2}{c}_{i}(x,\;t)}{\partial {x}^{2}},$$

where the effective transport velocity *v*_eff_(*x*) and diffusion *D*_eff_(*x*) account for varying pore diameter and Taylor dispersion^32,62^ and *c*_*i*_ is the pore cross-sectional averaged concentration. To predict transport dynamics through a junction block for a given flow, we determine exact expressions for the mean first passage time to exit the junction block via the outgoing parallel ${{{{{{{{\rm{pore}}}}}}}}}_{\parallel,{{{{{{{\rm{out}}}}}}}}}$ or the perpendicular ${{{{{{{{\rm{pore}}}}}}}}}_{\perp }$ in the spirit of ref. 63. To this end, we solve the pore junction geometry explicitly in the temporal Laplace space, see supplemental theory section 1.4. The algebraic solutions for the Laplace transformed concentrations determine directly the statistical moments of the mean first passage time to exit through the parallel outflow or the perpendicular pore. From these, we conclude on the variance of the transit time through a junction block, see supplemental theory section 1.5. In particular, from the ratio of probabilities to exit through the parallel versus the perpendicular pore, we conclude that transport is primarily governed by advection in parallel pores. As a consequence, contributions of the parallel outflow pore are dominant for both the total mean and variance of the transit time through a pore junction. Importantly, we take from this analysis, that traversing a pore junction can be interpreted as a stochastic process on the microscopic level of connected pores, whose statistical moments we can fully determine if the flow splitting within the junction is given, i.e. {*Q*_∥,in_, *Q*_∥,out_}.

Yet, as different pore junctions have different flow splitting, an additional stochastic contribution from sampling different pore junctions is superimposed for traversing across an entire porous medium. The impact of the superposition of two stochastic contributions stemming from different spatial scales on the variance in travel times is captured by the law of total variance,

2 $${{{{{{{\rm{Var}}}}}}}}[{T}_{\parallel,{{{{{{{\rm{out}}}}}}}}}]={{{{{{{{\rm{E}}}}}}}}}^{{{{{{{{\bf{f}}}}}}}}}[{{{{{{{{\rm{Var}}}}}}}}}^{{{{{{{{\bf{p}}}}}}}}}[{T}_{\parallel,{{{{{{{\rm{out}}}}}}}}}|\{{Q}_{\parallel,{{{{{{{\rm{in}}}}}}}}},{Q}_{\parallel,{{{{{{{\rm{out}}}}}}}}}\}]]\\+{{{{{{{{\rm{Var}}}}}}}}}^{{{{{{{{\bf{f}}}}}}}}}[{{{{{{{{\rm{E}}}}}}}}}^{{{{{{{{\bf{p}}}}}}}}}[{T}_{\parallel,{{{{{{{\rm{out}}}}}}}}}|\{{Q}_{\parallel,{{{{{{{\rm{in}}}}}}}}},{Q}_{\parallel,{{{{{{{\rm{out}}}}}}}}}\}]].$$

Here, the superscript **f** refers to the statistics of flow splitting in pore junctions, and the superscript **p** refers to transit time statistics through a pore junction unit. Note that E^**p**^[*T*_∥,out_∣{*Q*_∥,in_, *Q*_∥,out_}] and Var^**p**^[*T*_∥,out_∣{*Q*_∥,in_, *Q*_∥,out_}] follow directly as algebraic expressions from the first passage time through a junction block. Importantly, we understand these two expressions as functions of the random variables {*Q*_∥,in_, *Q*_∥,out_}. This allows us to link the pore junction transit time statistics with flow statistics. For low and intermediate disorder, we can account for the statistics of flow splitting in pore junctions explicitly. In these disorder regimes, the flow distribution is a multivariate Gaussian centred around the average flow *Q*_0_, see Fig. 3b. Furthermore, we found its variance to be growing linearly with disorder, *σ*_1_ = *α*_1_*χ*, *σ*_2_ = *α*_2_*χ* along the axes *q*_1_ = (*Q*_∥,in_ + *Q*_∥,out_)/2*Q*_0_ and *q*_2_ = (*Q*_∥,in_ − *Q*_∥,out_)/2*Q*_0_. Given these closed expressions for the flow statistics, we approximate the mean and variance of the transit time by a series expansion in the deviation from the average flow *Q*_0_, see supplemental theory section 1.6. Importantly, we can calculate all expressions up to any desired order explicitly. We collect terms respective to their order in *χ*^*k*^ and define the prefactors Λ_*k*_. We obtain a polynomial approximation on how the total variance of the first passage time depends on the heterogeneity of flows in the porous medium. Due to the Gaussian statistics of the flow rate splitting, odd powers vanish, Λ_1_ = 0 and Λ_3_ = 0. We can directly assess Λ_0_ > 0, Λ_2_ ≪ 0, and Λ_4_ > 0 for high Péclet numbers Pe_⊥_ ≫ 1, see supplemental theory section 1.6. Thus, the impact of porous media disorder *χ* on the variance of the first passage time across a porous medium yields

3 $${{{{{\rm{Var}}}}}}[{T}_{\parallel,{{{{{{{\rm{out}}}}}}}}}]\; \approx \;|{{{\Lambda }}}_{0}|-|{{{\Lambda }}}_{2}|{\chi }^{2}+|{{{\Lambda }}}_{4}|{\chi }^{4}.$$

**Fig. 4:**
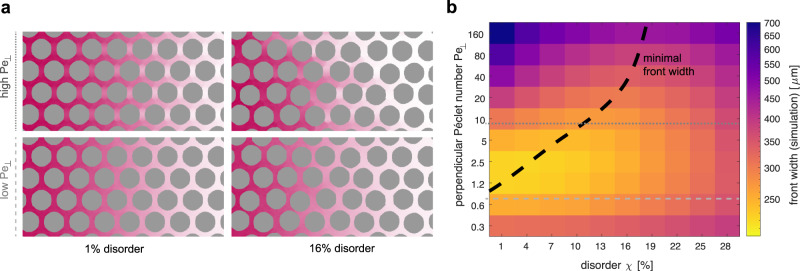
Non-monotonic dependence between dispersive front width and disorder vanishes for small Péclet numbers. **a** Illustration on how dispersion dynamics depend on advective transport. Simulation snapshots visualising how the sharpening of the dispersive front width relies on the transport mode of advection. A sharpening of the front width is only observed for large Péclet numbers. Porous media disorder has only little effect for low Péclet numbers, where transport in all pores is dominated by diffusion. **b** Evaluation of the effect of disorder and Péclet number on disperive front width. The effect of disorder and Péclet number on the dispersive front width are analysed in simulations. The black dotted lines indicates the position of minimal front width for varying Péclet number. Grey dotted lines indicate the Péclet numbers used in (**a**). In the simulation the Péclet number was altered by varying the molecular diffusivity *k*. In agreement with the theoretical prediction, the non-monotonicity is only apparent for high Péclet numbers Pe ≫ 1. Also, the disorder showing the sharpest dispersive front for a respective Péclet number increases with increasing disorder, which is in qualitative agreement with the analytical argument.

We predict for large Péclet numbers the variance of the first passage time through an entire porous medium to initially decrease with increasing disorder and to increase only for large disorders. Albeit not mathematically speaking an exact mapping, the variance of the first passage time is a direct predictor of the width of the dispersive front and the polynomial approximation shows an excellent collapse onto the data in Fig. 1c, see also supplemental theory section 1.8. Matching the Péclet number Pe_∥_ ≈ 30 and approximating Var^**p**^[*T*_∥,out_∣{*Q*_∥,in_, *Q*_∥,out_}] and E^**p**^[*T*_∥,out_∣{*Q*_∥,in_, *Q*_∥,out_}], we correctly predict the order of magnitude of the initial non-monotonicity with a dip of ${\chi }_{\min }^{{{{{{{{\rm{t}}}}}}}}}/{\chi }_{0}^{{{{{{{{\rm{t}}}}}}}}}\; \approx \;80\%$ at a disorder of ${\chi }_{\min }^{{{{{{{{\rm{t}}}}}}}}}=19.5\%$ in agreement with the fit to the data yielding ${\chi }_{\min }^{{{{{{{{\rm{f}}}}}}}}}/{\chi }_{0}^{{{{{{{{\rm{f}}}}}}}}}\; \approx \;83.5\%$ and ${\chi }_{\min }^{{{{{{{{\rm{t}}}}}}}}}=15\%$, see supplemental theory section 1.7. Analogous analysis for low Péclet number, Pe_⊥_ ≪ 1, predicts the initial decrease in variance with disorder to vanish, see supplemental theory sections 1.7, 1.8 and Supplementary Fig. S5. This is in agreement with extended simulations, see Fig. 4.

Importantly, our analysis offers the possibility to understand the physical origin of the initial sharpening of the dispersive front. We find that the variance a particle picks up from moving through a pore junction is on average reduced as the porous media’s disorder is increased. Specifically, the variance in the first passage time of traversing a junction block is maximal if there is no flow in the perpendicular pore, but decreases both if there is negative or positive flow in the perpendicular pore. The low flow velocities in perpendicular pores allow long diffusive meanderings of particles in the finite perpendicular pores before entering any of the neighbouring parallel pores. In particular, particles might take exploration tours in the low flow perpendicular pores and return to the junction splitting point instead of exiting the pore on the opposite side; this is an effect that cannot be accounted for, if back diffusion between pore bodies is neglected. Increasing the flow in perpendicular pores reduces the probability of initiating exploration tours through perpendicular pores and reduces the average transition time. To confirm this explanation more generally, we also ran simulations on a square lattice, for which we predict the physical intuition explained above to remain valid. Comfortingly, we also observe for the square topology a reduction of the dispersive front, see Supplementary Fig. S8. Overall, we demonstrated that transport statistics in the junction space is a powerful tool to generate intuitive insight on fluid flow-based transport in porous media. The presented concept can easily be modified and applied to a variety of morphologies and different types of porous media.

## Discussion

Investigating how porous media morphology optimises transport efficiency, we discover at first sight counter-intuitive transport characteristics that arise from the interplay of advection and diffusion in the heterogeneous flow fields of porous media. Performing a conceptual shift by considering transit time statistics on the level of pore junctions instead of individual pores solves the conundrum of our experimental data. Algebraically deriving the transport statistics of the junction space allows us to connect microscopic dynamics with macroscopic transport properties through entire porous media. This way, we link changes in the morphology of porous media with changes in the transport dynamics. Specifically, we can incorporate the heterogeneity of flow fields effectively into a macroscopic description of transport and asses the efficiency of transport through the whole porous medium. While this approach is especially suited for low to intermediate disorder *χ* < 30%, see Fig. 1c, the prediction at higher disorder values fails, as deviations from Gaussian flow rate profiles arise, which are not captured by our expansion, see Fig. 3 and Supplementary Fig. S9. The deviations from Gaussian flow rate profiles indicate that for high disorder, *χ* > 30%, the analysis needs to be adjusted to include correlations between junction units. In this context, the analysis of the variation in the dispersive front perpendicular to the pressure axis could help to disentangle correlations among pore junctions extending beyond nearest-neighbour correlations.

In our work, we present how introducing disorder increases the efficiency of transport. Intriguingly, a constructive role of noise in general arises in the broad context of transport processes^64–68^, with noise increasing transport efficiency in a seemingly unrelated system^54^, hinting to a deeper connection and strengthening the importance of comparing transport dynamics in vastly different systems to gain new perspectives on the constructive role of noise in transport dynamics. In conclusion, for the rich class of transport processes in porous media, the conceptual shift to pore junctions as the building blocks of porous media offers a change in paradigm for the multitude of applications of porous media from oil recovery, chemical reactors, and biological systems to the design of novel materials such as self-organised porous media and soft robotics.

## Methods

### Experimental methods

Microfluidic micromodels were produced following soft lithography methods in line with those detailed elsewhere^15^. The porous media were designed using circular obstacles of radius *r* = 60 μm on a hexagonal grid of lattice spacing *a* = 162 μm, so that the packing fraction was 0.5. Each obstacle was displaced from its grid location in a random direction by a distance chosen from a uniform random distribution up to a maximum of *χr*, with the constraint of a 10 μm minimum distance between obstacles. We use a chrome-quartz photomask and a negative photosresist (SU8 3025) to manufacture reusable templates in six designs with *χ* between 0.01 and 0.56. Poly(dimethylsiloxane) (PDMS) is poured over these templates, degassed under vacuum, and cured for 2 h at 75 °C, then the solidified layer is cut off and inlet/outlet holes are punched. The imprinted PDMS and a glass slide are primed in an oxygen plasma and adhered to one another, forming a microfluidic chip containing solid pillars as obstacles, separated by open channels with a thickness of 60 ± 3 μm. For each experiment, a chip was filled with deionised water, sonicated under flow to detach any air bubbles^69^, and then fluorescein-dyed water was fed into the inlet of the water-saturated chip from a syringe pump. For fluorescent imaging, filter cube 38 HE (excitation 470/40 nm, emission 525/50 nm (wavelength/bandwidth)) was used. We used flourescein at a concentration of 150 μM, which is within the linear range of a calibration curve made of pixel-intensity vs. concentration.

### Numerical methods

To simulate the spread of a diffusive solute in a porous medium, a Crank-Nicolson routine was employed^70^. To this end, the porous media used for the simulation followed the same design as the microfluidic micromodels: circular obstacles on a hexagonal grid were randomly displaced from their grid locations, as detailed in the ‘Experimental methods’. The porous medium was interpreted as a network of pores through which both advective and diffusive transport were considered. The simulation routine involved three major steps. First, the pores’ network structures were extracted by skeletonization of the designs used for the microfluidic experiments. Along the extracted skeleton, the minimal distance to the nearest obstacle wall was computed, to extract the varying radius along the pores. The hydraulic resistance along each pore was computed under the assumption of a slowly varying pore radius. Next, Kirchhoff’s circuit law was employed, conserving fluid volume at every network node to compute the flow profile in the network. At the left and right sides of the network, inlet and outlet pores were identified. A constant pressure drop was imposed across the medium, by setting the pressure *P*_in_ at every inlet pore and the pressure *P*_out_ at every outlet pore. Flow rates in pores were computed by a matrix inversion solving Kirchhoff’s circuit laws. The varying flow velocity along the pores was computed by dividing the flow rate through a pore by the cross-section varying along the pore and a Poiseuille profile was imposed. In a third step, the network topology and the flow profile were used to numerically integrate the advection–diffusion equation along individual pores using a Crank-Nicolson integration routine. To minimise numerical artifacts, pores were approximated as tubes with varying circular cross-sections. For the Crank-Nicolson integration routine, the dynamics are reduced to the spread along the skeleton and an effective Taylor dispersion accounting for varying boundary conditions is employed^32^. At junction points, merging and splitting conditions were implemented analogous to the Crank-Nicolson routine along individual tubes^70^. To minimise boundary effects at the inlet and outlet of the porous medium, inlet pores and outlet pores were identified and then artificially elongated by a factor of four. This is in contrast to the experimental routine, where an open area before and after the porous medium was created, which would cause numerical artifacts during skeletonization. A fixed concentration, *C*_0_ = 1, was set for inlet pores and at outlet pores an open outflow condition was implemented, which estimated transport by advection and diffusion across the last spatial simulation point. To simulate cases of different Péclet numbers, the diffusion constant was altered instead of the flow velocity, as this granted better numerical stability. Finally, the simulation was validated against analytical solutions in simple network geometries.

### Particle image velocimetry (PIV)

Flow velocity fields were extracted by particle imaging velocimetry (PIV). Fluorescent 1 μm polystyrene microspheres (FluoSpheres, ThermoFisher Scientific) were used as tracers, diluted to a 1:3750 volume ratio and pumped through the microfluidic chips at 100 μl/h. Fluorescence imaging was done at 18.18 fps with a 5 ms exposure and a 63 HE filter cube (excitation 572/25 nm, emission 629/62 nm (wavelength/bandwidth)) at 2048 × 2048 pixel resolution; typical flow speeds were 1–10 pixels/frame. A median image made from each image sequence was used for background subtraction. A white-light image of each experimental configuration was used to generate a mask of the obstacle locations. We used PIVLab^71^ to analyse the median-subtracted, masked image sequences, using default settings and successive interrogation areas of 64/32/16/8 pixels. The reported velocity maps are frame averaged over sequences of ~300 frames.

Having extracted the velocity vectors from the PIV analysis, flow rates were extracted using methods adapted from Alim et al.^35^. To this end, we took the flow velocity vectors on the skeleton of the pore space, and found the component of these flows projected along the skeleton. The flow rates in each pore were computed by dividing the projected vectors by the cross-sectional area varying along the pores. To avoid errors close to pore junctions, only the center third of each pore was considered. The median flow rate in each pore was used as a representative value, to reduce the sensitivity of the analysis to artificial outliers. By mapping flow rates to the skeleton, parallel and perpendicular pores are distinguishable.

### Estimation of the Péclet number

The Péclet number is defined as ratio of the timescale of advection to the timescale of diffusion, Pe = *τ*_adv_/*τ*_dif_, and characterises which mode of transport is dominant in a given setting. The advective timescale is computed using the characteristic flow velocity *U* over a characteristic length scale *l* as *τ*_adv_ = *U*/*l*, while the diffusive timescale is estimated over the same length scale with *τ*_dif_ = *D*/*l*^2^, resulting in Pe = *Ul*/*D*. However, spatial heterogeneity leaves ambiguity in the definition of the characteristic velocity. As such, we define two different Péclet numbers to effectively capture the essential transport dynamics in the porous medium. These are the parallel and perpendicular Péclet numbers, Pe_∥_ and Pe_⊥_, which relate to the flow statistics parallel and perpendicular to the pressure axis, respectively. Specifically, for Pe_∥_ the average flow velocity parallel to the pressure axis is taken as the characteristic flow velocity while Pe_⊥_ considers the flow perpendicular to the pressure axis. In both cases, the same diffusivity *D* and the characteristic length scale of a (diffusive) pore are considered. While Pe_∥_ is accessible through experiments, Pe_⊥_ gives a more insightful estimate in line with the theoretical considerations. If detailed knowledge of the flow statistics is available, both Péclet numbers can be converted into each other. We use porous media with an intermediate degree of disorder as a benchmark to estimate Pe_⊥_. Specifically, using the numerically computed flow fields, we find that Pe_∥_/Pe_⊥_ ≈ 5.

For the microfluidic experiments, the parallel Péclet number is estimated using the literature value^72^ of *D* = 4.25(1) × 10^−6^ cm^2^ s^−1^, and *U* ≈ 350 μm s^−1^ by measuring the propagation of the dispersive front between two recorded frames. As a characteristic length scale, the size of a diffusive pocket was estimated as *l* ≈ 40 μm. Here, rather than the lattice spacing between the pore centres, the average length of the pore throats was estimated. For this, from the pore length, which estimates half the lattice spacing, the average perpendicular pore diameter at junction points was subtracted. This yields a Péclet number of Pe_∥_ ≈ 30 for the experiments.

### Measurement of the dispersive front width

While Fig. 2 characterises the spatial profiles of both the numerical simulations and the experimental data, the form of the data is different. To extract the maximum information from both the simulation and the experiment, different estimates of the dispersive front were employed, yet both metrics have the same functional dependency on the effective diffusion constant (i.e. as measures of dispersion), see also supplemental theory section 1.8.

#### Numerical simulation

Detailed concentration values along the individual pores are available for the numerical simulation. To compute the width of the dispersive front, a projection of these data onto the pressure axis is performed. For this, the mean concentrations of individual pores, weighted by their pore cross-sectional areas, are projected onto the pressure axis, creating an averaged profile. To avoid artifacts from the boundary walls, only the middle half of the porous medium was considered for the analysis. To compare simulation runs with different degrees of disorder, profiles were evaluated at the time when the point mid-way between the inflow and outflow tubes *x*_*e*_ was first reached by half the saturation concentration, *C*_0_/2, of the averaged profile.

As a measure of the width of the dispersive front the deviation from a step function profile was quantified. After normalisation by *C*_0_, the first half of the front profile, *C*(*x*) ∀ *x* < *x*_*e*_, was flipped to $\bar{C}(x)=1-C(x)$, creating a new profile that is sharply peaked around a maximum of $\bar{C}({x}_{e})=1/2$. As a measure of the front width, the integral ${I}_{\bar{C}}$ below the modified profile $\bar{C}(x)$ was evaluated, which scales proportionally to the root of an effective diffusion constant, ${I}_{\bar{C}}\propto \sqrt{{D}_{{{{{{{{\rm{eff}}}}}}}}}}$.

#### Experimental data

While evaluating the dispersive front width from experimental data, care was taken to minimise the effects of noise on the signal. The analysis was conducted in matlab. An image taken prior to injecting any dye was used for background subtraction, which was applied to all later recorded images. To correct for non-homogeneous illumination, the fluorescence images were then normalised by the background-corrected, last recorded frame, corresponding to full saturation. A mask of pillar locations was made by thresholding the last recorded frame and positioning circular mask elements at the centroids of the obstacles’ silhouettes. The masked pillars were assigned with the scalar representation of not a number, nan. As with the simulations, to avoid boundary effects, only a 4.3 mm wide window around the middle axis of the chip was considered. Images were filtered with a Gaussian filter of width 3 pixels, where nan assigned values were excluded from the analysis, by using nanconv^73^. A projected intensity profile was computed from the normalised, cropped, and filtered images as the mean value perpendicular to the pressure axis, excluding the masked obstacles. The resulting profile varies between the relative intensities of 0 (undyed) and 1 (fully dyed).

The integral ${I}_{\bar{C}}$ gives a precise characterisation of the front width for the numerical simulation, but is sensitive to fluctuations or distortions in the tails of the profile, including the weak, but observable, effects of bleaching in the experiments. As such, experimentally the width of the dispersive front is directly evaluated from the length, *w*, over which the normalised intensity profile is between 0.25 and 0.75. As an infinitely sharp step of the initial concentration is not realisable due to diffusive spreading in the tubing before entering the chip, we record the equivalent front width, *w*_0_, when the dye front first enters the obstacle area. The front width is then characterised 15 s later, to ensure consistency across experiments, by the measurement of *w*_*c*_ = (*w* − *w*_0_). In a final step, the average offset 〈*w*_0_〉 is added as a constant offset to each measurement point to match the order of magnitude of the observed front width in the microscopy images. While this method is designed to minimise the effects of numerical artifacts, we demonstrate that the non-monotonic dependency of *w*_*c*_ on *χ* is robust to changes in the choice of evaluation parameters, see Supplementary Fig. S6.

## Supplementary information

Supplementary Information

## Data Availability

The collected experimental data, simulation results, and source data underlying Fig. 1c and Fig. 3b are available in the mediaTUM repository under 10.14459/2022MP1684458.

## Code availability

A Mathematica notebook allowing the tractability of calculations, code for generation of the porous media design and the integration routine to simulate dispersive spread in porous media are deposited also in the mediaTUM repository under 10.14459/2022MP1684458. Plotting routines and further code snippets are available from the corresponding author upon reasonable request.
