# Supplementary material for: Dispersive transport dynamics in porous media emerge from local correlations
Source: Nat Commun. 2022 Oct 6;13:5885. doi: 10.1038/s41467-022-33485-5 (PMC9537155; doi:10.1038/s41467-022-33485-5)
Supplement: Supplementary file 1 — Supplementary Information [file 41467_2022_33485_MOESM1_ESM.pdf]

Dispersive transport dynamics in porous media emerge from local correlations

Supplementary Material

Felix J. Meigel, Thomas Darwent, Leonie Bastin, Lucas Goehring, Karen Alim

September 11, 2022

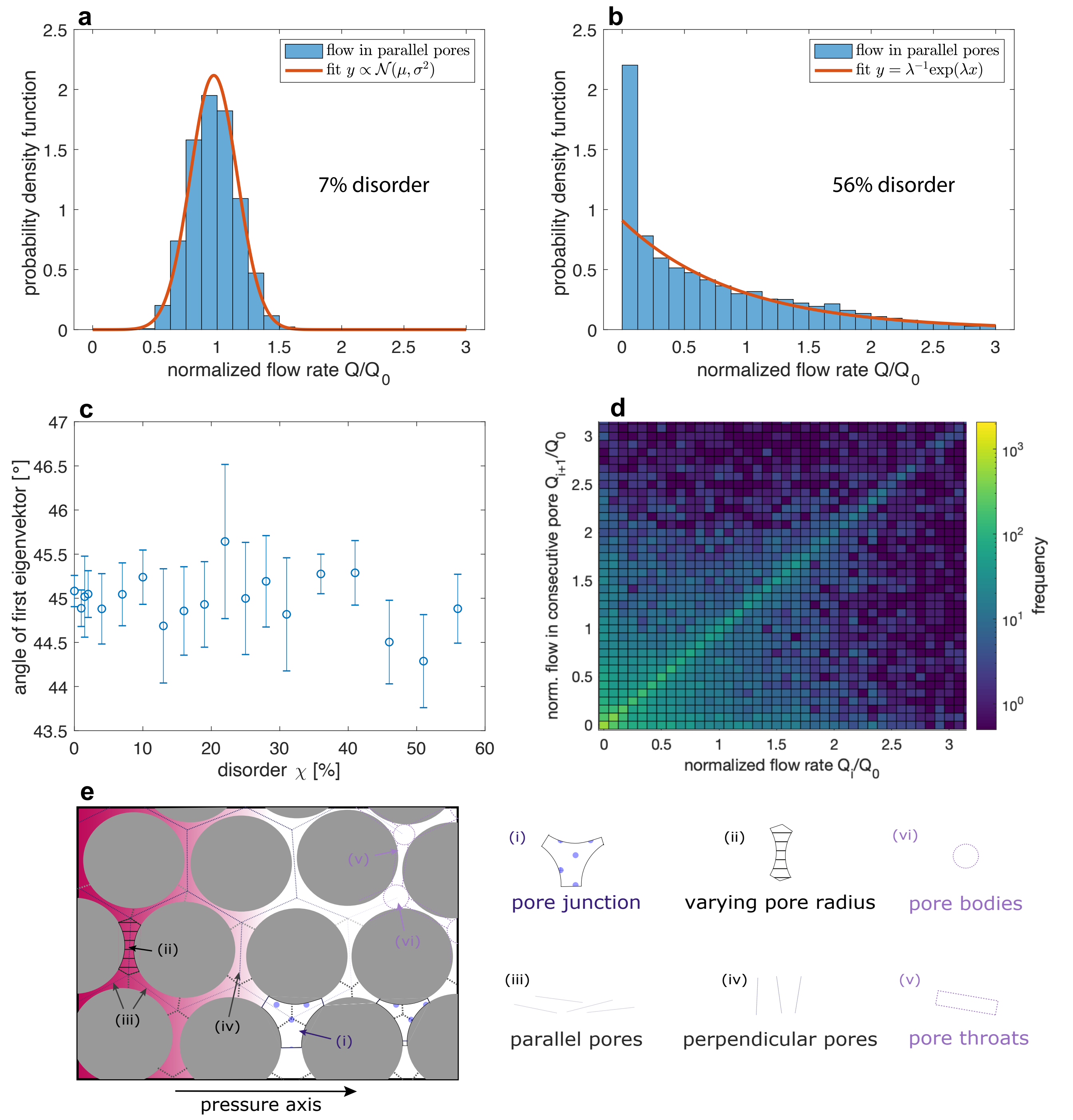

Fig. S1. Additional Flow Statistics. (a,b) Pore Space Flow Statistics for Intermediate and High Disorder. Intermediate and high degrees of disorder show different pore space flow statistics. Here, only parallel pores are taken into account. While for intermediate degrees of disorder the flow statics are well approximated by Gaussian statistics, high degrees of disorder are approximated by an exponential decay. Fitting parameter used are $\mu = 0.973$, $\sigma^2 = 0.036$, and $\lambda = 1.1$. **(c) Orientation of the Eigenvector of the Mulitvariate Gaussian Fit to the Junction Statistics** We find that the orientation of the eigenvector of a mulitvariate Gaussian fit to consecutive parallel pores is independent of disorder. **(d) Flow Statistics of Consecutive Pores** The statistics of flow in consecutive pores for a high degree of disorder (65% degree of disorder) is plotted analogously to the plots in Fig. 3 in the main manuscript. Note, that for high disorder a structure of three clusters cannot be identified. **(e) Cartoon depicting pore junction definitions** The definition of pore junctions, pore throats, and pore bodies are contrasted against each other.

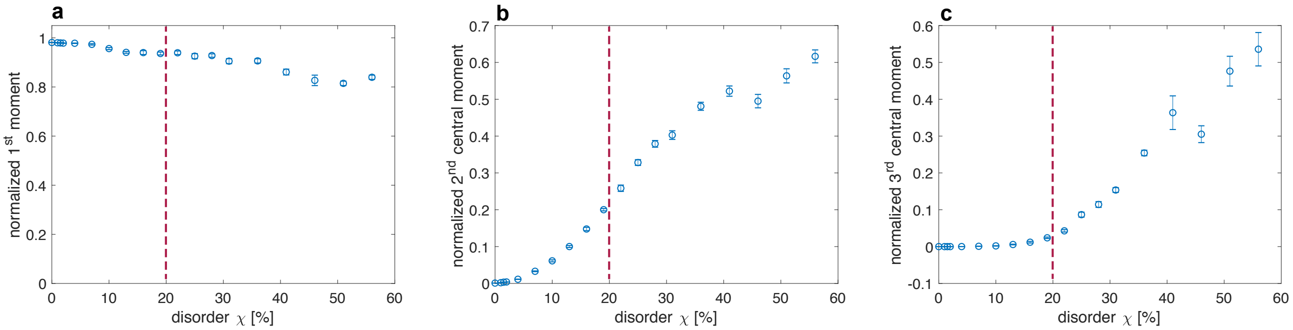

Fig. S2. Central Moments of the Pore Space Flow Statistics. The statistics of the first **(a)**, the second **(b)**, and third central moment **(c)** of the pore space flow statistics over parallel pores give insight up to which disorder a Gaussian approximation is justified. A dotted red line indicates that up to 20% disorder, where the third central moment is vanishing. We consider Gaussian statistics to work well up to 20% disorder.

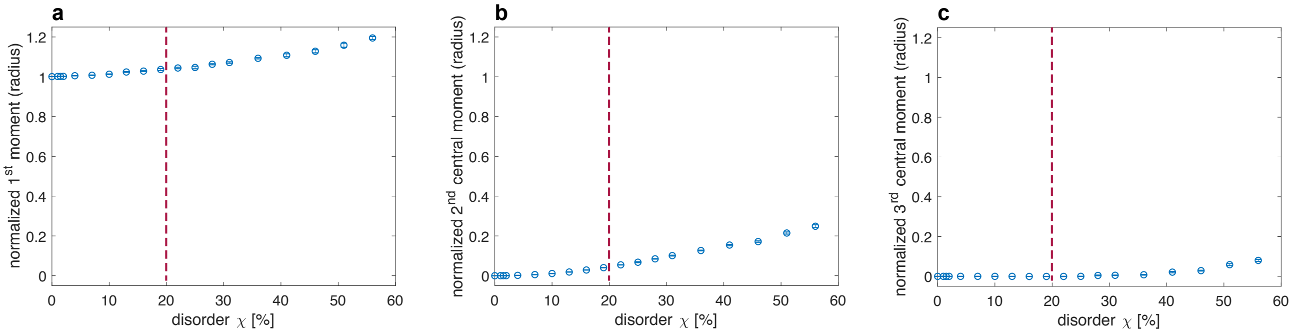

Fig. S3. Radius Statistics. The statistics of the first **(a)**, the second **(b)**, and third central moment **(c)** central moment of the pore space radius statistics give insight how much radius variation are affected by disorder. We find that variations of the radius are smaller than variations in the flow statistics and that up to a disorder of 20% radius variations are negligible.

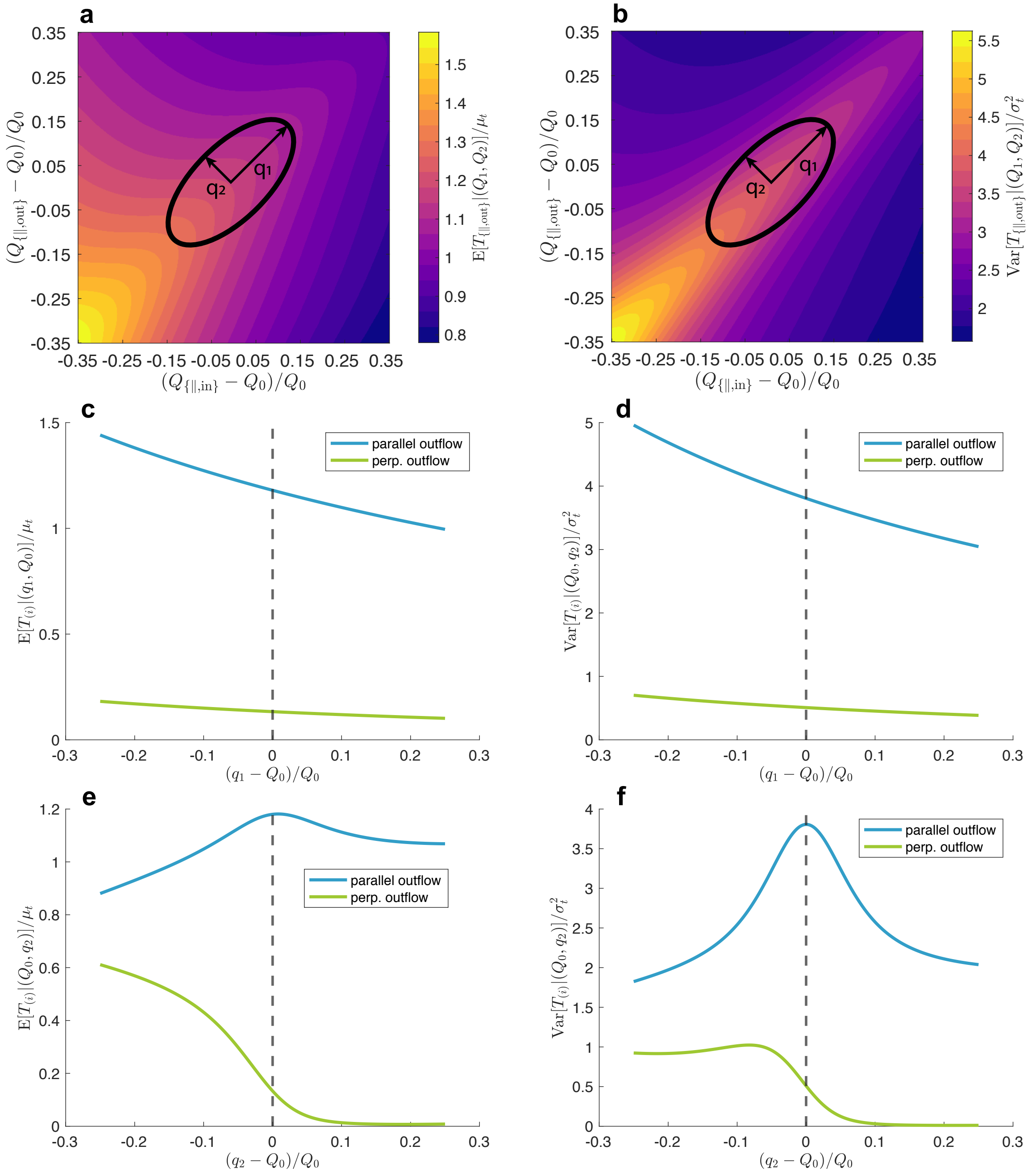

Fig. S4. Dynamics of Transport Through Pore Junction Units. Here, we exemplify the transport dynamics through pore junction units: (a,b) show maps analogous to the junction maps in flow statistics, (c,d,e,f) show the dynamics along the eigenvectors q_1, q_2. (a) shows the mean passage time to exit through the parallel outflow pore for different flows Q_1, Q_2 in the parallel inflow and the parallel outflow pore, respectively. The error ellipsoid indicates the gaussian flow statistics. Here, the variance over the function with respect to the junction statistics is to be computed for the total variance. (b) shows the variance of the passage time through the junction block with exiting through parallel outflow pore for different flows Q_1, Q_2. Here the mean over the function with respect to the junction statistics is to be computed for the total variance. In (c,d,e,f) we denote the dynamics of exiting through the parallel outflow pore with blue and the perpendicular pore with green. Graphically estimates of the first and second derivatives can be obtained. Specifically, we find that the variance of the passage time for particles exiting through the parallel outflow pore has a strong negative second derivative along q_2 if evaluated at (Q_0, Q_0). Contrasting eigenvector q_1 and q_2, we find that the transport dynamics only weakly change along q_1 but strongly change along q_2.

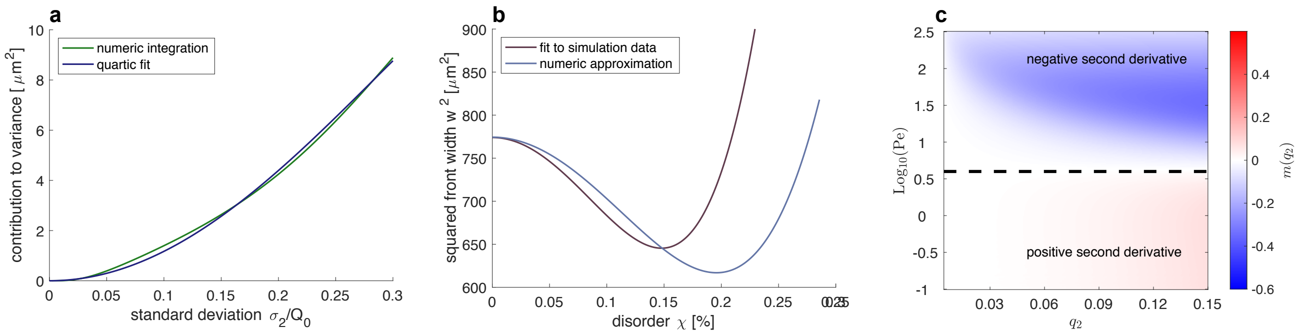

Fig. S5. Semi-Numeric Analysis of the Non-Monotonicity and the Effect of Varying Péclet Numbers. **(a) Visual Example of Fitting Quality on Semi-Numeric Integration Routine** Here, the contribution stemming from $\mathrm{Var}^{\mathbf{f}}[\mathrm{E}^{\mathbf{p}}[T_{\|,\mathrm{out}}|\{Q_0, q_2\}]]$ is visualised. The parameters of the quartic fit are shown in table S1. **(b) Agreement Between Fit on Full Numeric Simulation with Prediction From Semi-Numeric Analysis.** While the numeric simulation models the dispersion through a porous medium, the semi-numeric result is based on the analytic calculation of transit time moments through a pore junction unit. Here, the semi-numeric results only shows an approximation taking the variation of q_2 into account. A more refined analysis will shift to smaller dips with lower minimal disorder. A detailed description of the procedure underlying this figures is given in section 1.7. **(c) Mathematical Analysis Demonstrates Non-Monotonicity to Vanish For Small Péclet Numbers.** The change in the mean variance of leaving the parallel outflow pore is plotted as function of the variable q_2, that controls the flow splitting, see section 1.6. A negative second derivative is only obtained for large Peclet numbers $\mathrm{Pe} > \mathrm{Pe}_c$, indicated by a dotted line.

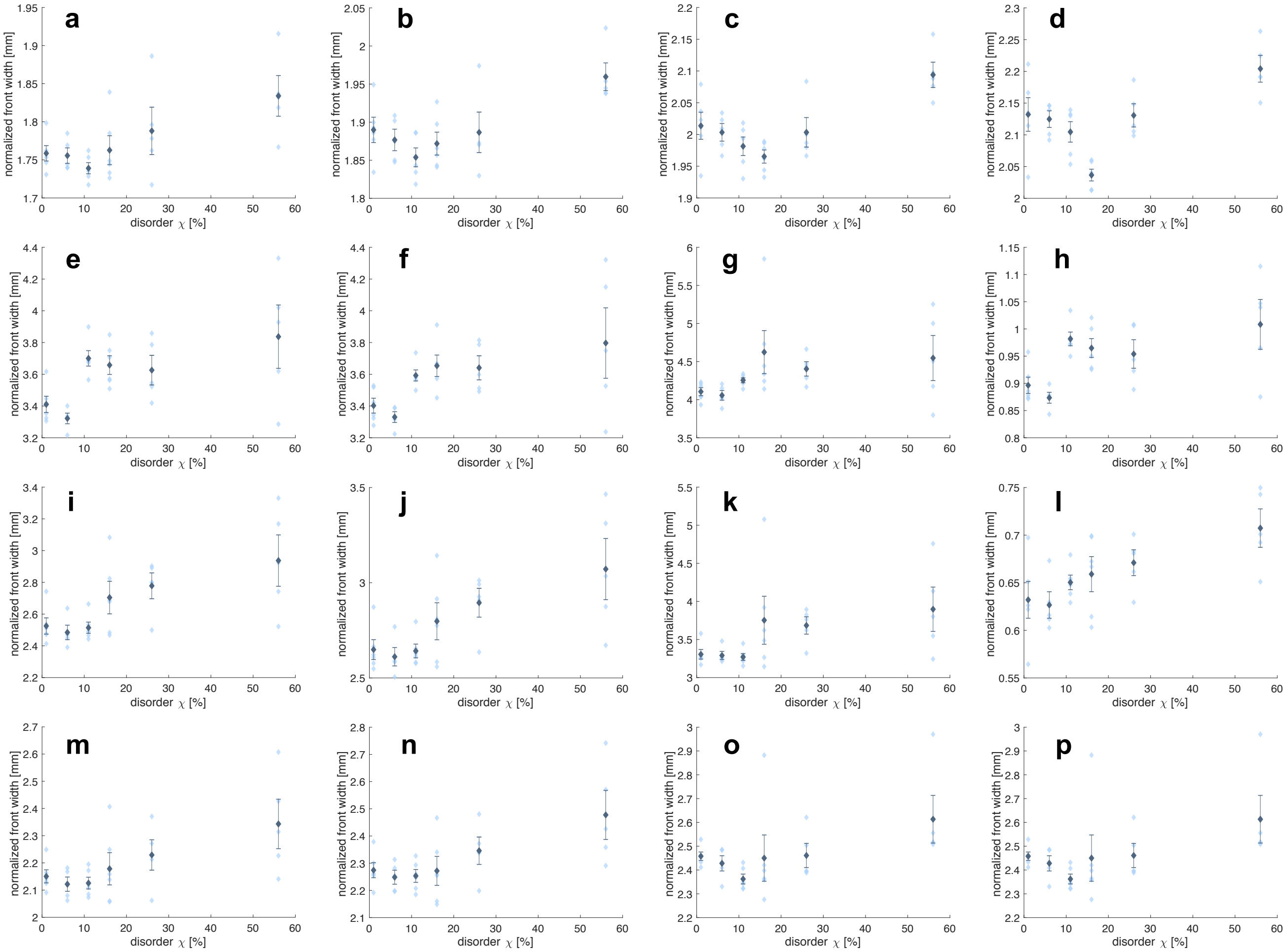

Fig. S6. Initial Decrease of Dispersive Front is Insensitive to Specific Parameter Choices Evaluation of the experimental profiles for different parameter choices. All parameter choices show the same trend of an initial decrease of front width with increasing disorder. For (a,b,c,d) the length between 25% and 75% disorder was evaluated. The profile 10 seconds after solute first reached into the pore space was recorded for normalisation. The analysis was obtained from the profiles recorded 5 s,10 s,15 s,20 s later for (a,b,c,d) where two consecutive frames were both considered for normalisation and analysis. For (e,f,g,h) no normalisation was considered, the profiles were recorded 25 s after the dye first reached into the porous medium and two consecutive frames were considered for analysis. The distance between $15\%-65\%$,$25\%-75\%$,$35\%-85\%$,$8\%-50\%$ were considered for (e,f,g,h) respectively. The analysis of (i,j,k,l) follows with the analogous parameters, but for normalisation the first frame of the solute reaching into the porous medium was used. Also the analysis of (m,n,o) follows the same analogous patterns, but the profile 5 s after the solute reached into the porous medium was considered for normalisation. (p) follow with the same parameter as (o), but here the frame after 30 s was considered for analysis.

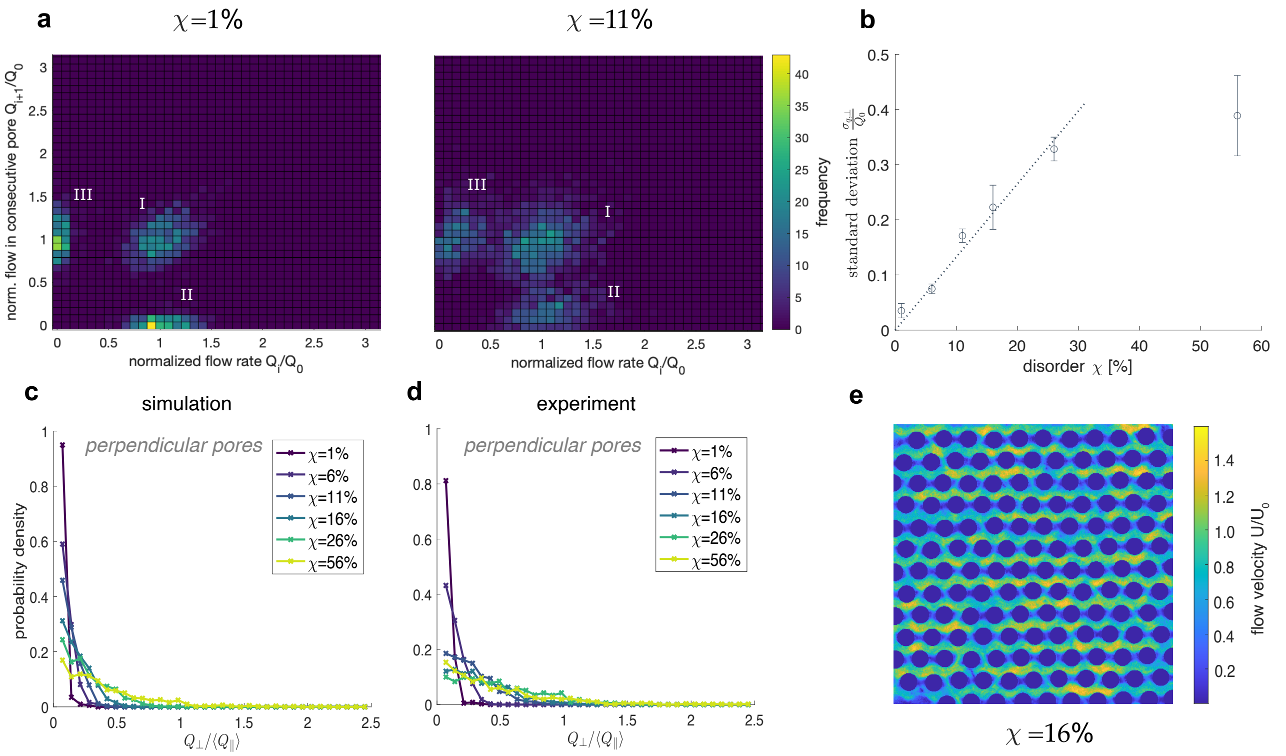

Fig. S7. Particle Image Velocimetry Analysis Shows that the Flow Statistics of Porous Media up to Intermediate Disorder are Captured by Gaussian Statistics. This figure gives the experimental analogue of the results in Figure 3 of the manuscript. The Particle Image Velocimetry (PIV) analysis is detailed in the manuscript methods. **(a) Flow Statistics of Consecutive Pores.** The statistics of flow in consecutive pores for low disorder ($\chi = 1\%$) and intermediate disorder ($\chi = 11\%$) are plotted. For low and intermediate degrees of disorder, three clusters are identified. The two clusters containing parallel and perpendicular pores increase in size with increased porous media disorder, in analogy to Fig. 3a in the manuscript. However, we noted finite size effects due to our sampling procedure in the statistics of parallel pores, as particles tracked through several consecutive parallel pores induce correlations, which decreases the effective sample size of the parallel pores. As all clusters contain the same information about the pore junction statistics–as detailed in the manuscript–further analysis focuses on the perpendicular pores. **(b) Effect of Disorder on Flow Statistics.** The standard deviation of the marginalized distribution of the perpendicular pores is plotted against the disorder of the porous media. Analogous to Fig. 3b, we predict a linear increase in the standard deviation with increasing disorder. A linear regression was performed in the range $\chi \in [6\%, 26\%]$ yielding a goodness of fit of $R^2 = 0.96$ with slope $m = 1.33$. **(c,d) Marginalized Distribution of the Flow.** Statistics of the perpendicular pores are given for (c) the simulations and (d) experiments. The flow distributions of both cases show excellent agreement, and the experimental and simulation data sets demonstrate how the variance increases with increased porous medium disorder. **(e) Velocity Field obtained by PIV Analysis.** Analogous to Fig. 2c in the manuscript, the flow velocity field obtained by PIV analysis for a porous medium with $\chi = 16\%$ is shown.

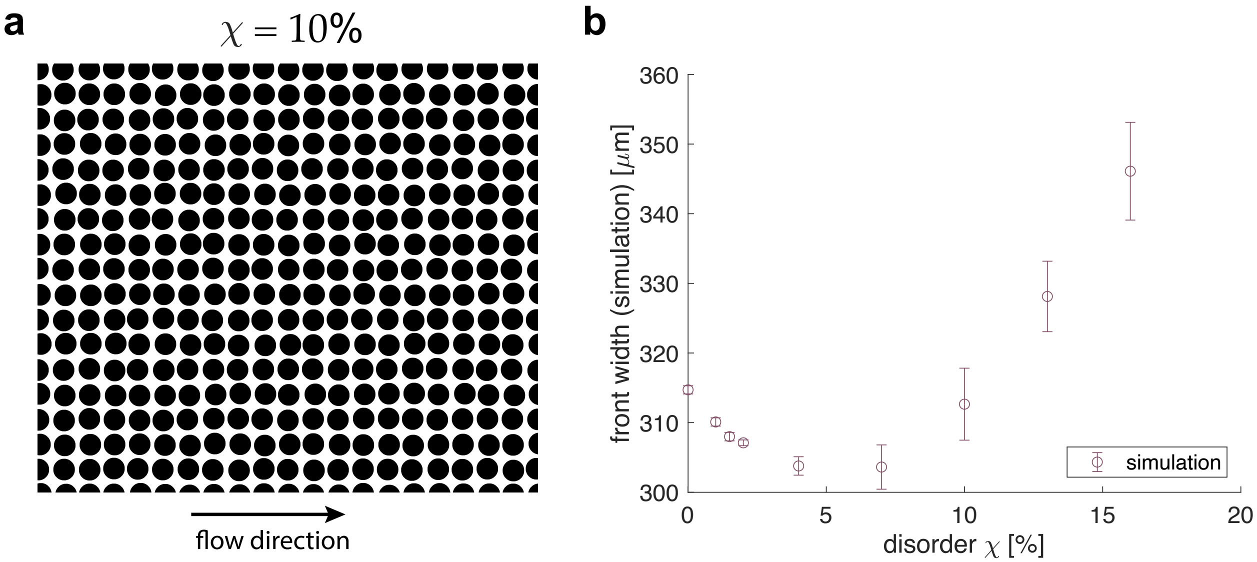

Fig. S8. Reduction of the Dispersive Front Width with Increasing Porous Media Disorder for a Square Lattice. This figure is analogous to Fig. 1c in the manuscript, but is based on a different lattice symmetry. **(a) Visualization of the Pore Space.** An example pore space for a porous medium based on a square lattice with disorder of $\chi = 10\%$ is shown. **(b) Change of the Dispersive Front Width with Disorder.** The dispersive front width is measured as a function of disorder for $\mathrm{Pe}_{\|} \approx 30$. Errorbars show the standard deviations computed over a sample size of $\mathrm{n} = 6$. The flow rate is adjusted to match the mean flow velocity of parallel pores. as in Fig. 1c. Notably, the square lattice shows more strongly varying pore radii than the hexagonal lattice, as disorder is added.

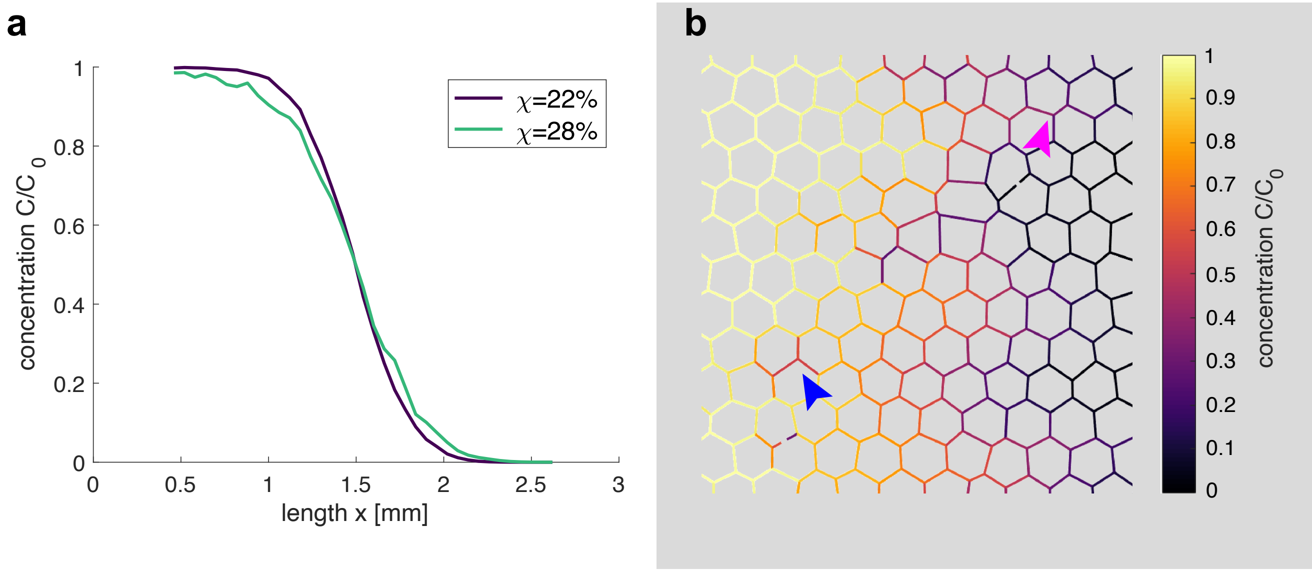

Fig. S9. Transition from Medium to High Disorder Qualitatively Affects the Dispersion Dynamics. (a) Projected Concentration Profiles (Simulation). The projected flow profiles are smooth in the tails up to disorders of approx. $\chi = 25\%$ and become visibly rough, or 'bumpy' for larger disorder. Two exemplary profiles were chosen, and the same simulation parameters as in Fig. 1c were used. The roughness indicates that a wider porous medium needs to be considered for smooth statistics, due to an increase in correlation between parallel pore junctions. **(b) Emergence of Correlations Between Pore Junctions.** A snapshot of the dispersion simulations shows the cause of these correlations in the emergence of high velocity 'streets' (pink arrowhead) and low velocity 'pockets' (blue arrowhead). To increase visibility, the average solute concentration within each pore is mapped to and shown along the skeleton of the pore space, only. High velocity 'streets' extend along several pores and locally advance dispersion. In low velocity 'pockets' the flow velocities of neighbouring parallel pores is small, so that those pockets lack solute until they are slowly filled by diffusion alone. Both structures are consequences of large-scale correlations between pore junctions.

1 Supplemental Theory

In this supplemental theory, we give a detailed derivation of the results presented in the main manuscript. We elaborate how heterogeneity in the pore space affects flow statistics and by this affects transport statistics through entire porous media. In particular, we explain how pore junction statistics allows understanding of how microscopic structures affect macroscopic transport properties. To this end, we give a detailed discussion about different aspects of the theory and the analytic methods presented in the manuscript. Finally, we connect this with intuitive explanations motivating the choice of methods employed. Foremost, this supplement should assist the understanding of the ideas presented in the manuscript and allows for translating the physical insight gained in this work to applications in different settings.

1.1 Efficiency of transport and connection to disperse front width

Characteristic for flow based transport is the presence of advection and diffusion as two different modes of transport. In simplistic 1-dimensional settings, the transport mode of advection dominates the mean transport dynamics and the transport mode of diffusion causes dispersion that manifest as deviations from the mean transport dynamics. While the interplay between advection and diffusion causes already in simple geometries - like 3-dimensional straight tubes with circular cross-section - intricate dynamics, a characterisation in terms of mean transport dynamics and deviations from the mean transport dynamics remains useful for application and analysis of technical and biological systems. Both complex technical and biological systems are often situated in temporally changing environments, on which the systems need to respond by the fulfilling of tasks or by adaptation. Crucial for the precise functioning of these systems is the response to changes in solute concentrations. While mean transport dynamics describe how fast chemical signals are transported through systems, the signal quality is described by deviations from the mean transport dynamics. Does the signal traverse through the systems with long, shallow concentrations gradients imposing the necessity to respond to small concentration changes, or does the signal propagate with a steep gradient allowing for a strong response?

Inspired by this idea, we define efficient transport through a system as transport that minimises deviations from the mean transport dynamics. For this, we quantify the deviation from the mean transport dynamics in experiment and simulation by measuring the width of a dispersive front. For the analytic calculations, we quantify the variance of the passage time dynamics. To connect the approaches, we make use of the law of large numbers to draw a proportionality between the standard deviation in the passage time statistics with measurements of the front width. Importantly, we don't restrict ourselves to Gaussian statistics neither in the experiment, the numerical simulations, nor the analytic calculations, but explicitly recognise the existence of higher order statistics in the transport through porous media, see also supplement section 1.8. Regarding the analytic approach, we want to emphasise already at this point, that the non-monotonicity we describe for the variance translates qualitatively to the standard deviation and hence the front width, as detailed out in section 1.7. In the next section, we will put a detailed focus on the qualitative behaviour of the dispersive front by visually comparing the spreading dynamics in experiment and simulation.

1.2 Qualitative effect of porous media disorder on dispersion dynamics

To corroborate our counter-intuitive experimental findings of a decrease in dispersive front width with increasing disorder, we perform numerical simulations of dye dispersion, see Fig. 2 a, b, c in the main manuscript and Methods. In our numerical simulations, the porous media is built of individual pores connected into a network, as previously established for porous media of high packing fraction [1, 7, 6]. Pores are implemented as channels of varying diameter. This approach allows us to capture the effect of Taylor dispersion in individual pores, while effectively reducing the complexity of a spatially extended structure. In good agreement with the experiment, we visually recover how an initially sharp front disperses in different manners depending on the degree of disorder. Moreover, numerically quantifying the front width by direct deviation from a sharp profile, we recover the experimentally observed non-monotonic behaviour of the front width as a function of porous media disorder, see Fig. 1 c in the main manuscript. This confirmation now raises the question how transport efficiency is linked to altered transport dynamics on the level of individual pores.

Zooming in onto the dispersion dynamics on the pore level, we distinguish pores with an orientation parallel and perpendicular to the pressure gradient, i.e. enclosing an angle of $\alpha < 45°$ or $\alpha \geq 45°$ with the axis of the pressure gradient, respectively. For low degrees of disorder (e.g. 1%), we observe in both experiments and simulations that dispersion dynamics are faster in parallel pores than in perpendicular pores with almost equal dynamics for horizontally parallel lanes of transport. Transport here resembles a ladder structure with fast dispersion along the ladder

stiles while ladder rungs take longer to reach full saturation. Yet, at intermediate degrees of disorder (e.g. 16%) the ladder structure disappears, instead perpendicular pores do not lack behind the saturation of neighbouring parallel pores and dynamics now vary between horizontally parallel lanes of transport. At even higher disorder (e.g. 56%) lanes of transport now traverse both parallel and perpendicular pores. Lanes split and merge enclosing extended regions of low dye concentration. While clearly different dispersion patterns are identified for different degrees of disorders, it is unclear how these patterns emerge and what their relevance for transport efficiency is if only the experimental findings are analysed.

Here, numerical simulations allow us to correlate different dispersion dynamics with the underlying flow fields in the respective porous media, see Fig. 2 b, c in the main manuscript. We find that lanes of transport coincide with lanes of large flow velocities. At low disorder, high flow velocities are limited to parallel pores. Yet, at intermediate disorder, flow velocities in perpendicular pores become comparable to those in parallel pores, whose velocity distribution itself now varies broadly. At high disorder, highways of high flow emerge traversing parallel and perpendicular pores. These highways enclose extended regions of low velocity, which coincide with low dye concentration. Taken together, we find that changes in the front width are closely connected to changes of the flow field through the porous medium. However, how to connect changes in the flow field with a non-monotonous change in front width and transport efficiency cannot be seen readily. To establish this connection, we turn to how transport through a pore junction unit is calculated. To this end, we first define the geometric structure of a junction block and then show how transport in an individual pore is described using an advection diffusion equation.

1.3 Pore junction building block

In porous media, pores with vastly different average flow velocities meet at pore junctions. To capture this effect, we take pore junctions as central building block, instead of individual pores. Refining the description of pore bodies by defining pore junctions, we combine the advantages of pore space statistics and pore bodies, see also Fig. S1 e. Pore space statistics have been proven to successfully capture the statistics flow rates in porous media, while pore bodies proved useful in the description of front propagation of biphasic flow. We combine pores meeting in a pore body to build pore junctions. Doing so, we define the transport statistics in pore-bodies in agreement with the pore-space statistics while accurately capturing the interplay between pores with different flow rates in pore bodies.

The definition of pore junctions demand geometric adjustments to account for the flow statistics in specified porous media. The definition of the pore junction building blocks need to effectively treat the problem of back-diffusion and be most simply represented in the flow statistics. Here, we describe the geometry of pore junctions building blocks best suited to the experimental design of the porous media under consideration in this work. Having identified that the statistics of pore junctions are fully captured by the statistics of cluster I in Fig. 3, we have a natural choice of the pore junction building block: The pore junction building block consists of a parallel inflow pore, a parallel outflow pore and a perpendicular joining these two pores.

We account for back diffusion in the parallel, high flow pores by considering the pores only over half their length $\ell/2$. Doing so, we avoid taking junctions as end points. Note, that we allow for back diffusion in these pores by virtually extending the inflow pores to $-\infty$. The concept of virtually extending the inflow pore to $-\infty$ is illustrated with a detailed example in section 1.9 We cannot apply the same definition to perpendicular pores, where back diffusion in the pore itself is a major contribution to transport efficiency. We thus consider perpendicular pores over the full length ℓ. To effectively treat the outflow point of these pores, we make the approximation that back diffusion into the perpendicular pore once a particle has entered a parallel, high flow pore is negligible. We account for a correction of the building block end point with geometric terms detailed out in section 1.6.

Alternative to defining pore junctions as a Y-shaped geometry, here considering pore junctions as H-shaped geometry is suitable, which recreates the ladder structure found for porous media with low pore space heterogeneity. Choosing this geometry, the problem of back diffusion in perpendicular pores is fully treated with no approximations needed. While demanding a more complicated analysis of the flow statistics, this building block choice gives the same qualitative insight like the Y-shaped building block [analysis not shown], and a posteriori justifies the assumptions made. Yet, as the H-shaped structure is specific to the porous media structure considered in this work, for improved readability the Y-shaped building blocks are discussed instead.

To understand transport dynamics through the junction block, we next review effective Taylor dispersion in pores with varying diameter. Based on this, we derive the transport dynamics in a pore junction block.

1.4 Effective advection and diffusion in pores with varying diameter and transport through junction blocks

Taylor dispersion describes the interplay between advection and diffusion in a straight cylindrical tube. Importantly, this analysis gives rise to an effective one-dimensional description based on an effective diffusivity. While the original analysis of Taylor is only applicable to tubes with constant radius, central manifold approaches proved as powerful tools to incorporate additional modifications, see [3] for a pedagogical review. Here we recapitulate the analysis of pores with varying circular cross-section $A(z)$, where we measure the length of the tube in z-direction. In analogy to the well-known Taylor-dispersion, we can write down

$$\begin{aligned}\frac{\partial c}{\partial t} &= -\left(v - \frac{k}{r^2}\frac{\partial r^2}{\partial z}\right)\frac{\partial c}{\partial z} + k\left(1+\frac{r^2v^2}{48k^2}\right)\frac{\partial^2 c}{\partial z^2} \\ &= -v_{\text{eff}}\frac{\partial c}{\partial z} + D_{\text{eff}}\frac{\partial^2 c}{\partial z^2},\end{aligned} \tag{1}$$

where c is the cross-sectional averaged concentration, k is the molecular diffusivity, r is the tube radius and v is the cross-sectional averaged flow velocity. We use this result for the numerical simulation of dispersive transport through porous media.

Next, we consider transport in a junction block. For this, we tile the entire porous medium into our minimal unit, which consists of two consecutive parallel pores denoted $\{\|, \text{in}\}$ and $\{\|, \text{out}\}$, respectively, joined with a perpendicular pore $\{\perp\}$, as described in detail in the previous section. The agreement of our numerical simulations with our experimental data already confirms that the transport dynamics is successfully captured by Taylor dispersion in channel-like pores of varying diameter connected in a planar network. Analytically, transport through an individual pore $i \in \{\|, \text{in}; \|, \text{out}; \perp\}$ is given by an advection-diffusion-equation:

$$\frac{\partial c_i(x,t)}{\partial t} = -v_{\text{eff},i}(x)\frac{\partial c_i(x,t)}{\partial x} + D_{\text{eff},i}(x)\frac{\partial^2 c_i(x,t)}{\partial x^2}, \tag{2}$$

where the effective transport velocity $v_{\text{eff}}(x)$ and diffusion $D_{\text{eff}}(x)$ account for varying pore diameter and Taylor dispersion [4, 3]. At a junction, all pores are constrained to have the same concentration $c_{\|,\text{in}}(\ell_{\|,\text{in}}, t) = c_{\|,\text{out}}(0,t) = c_\perp(0,t)$ and mass flux is conserved $A_{\|,\text{in}}(\ell_{\|,\text{in}})j_{\|,\text{in}}(\ell_{\|,\text{in}}, t) = A_{\|,\text{out}}(0)j_{\|,\text{out}}(0,t)+A_\perp(0)j_\perp(0,t)$, where $A(x)$ denotes the pores cross-sectional area and $j_i(x,t) = -v_{\text{eff},i}c_i(x,t) + D_{\text{eff},i}(x)\frac{\partial c_i(x,t)}{\partial x}$ the concentration flux. To predict transport dynamics for a given flow, we want to determine exact expressions for the mean first passage time to exit a junction via the outgoing parallel pore$_{\|,\text{out}}$ or the perpendicular pore$_\perp$ in the spirit of Ref. [5]. For reasons of readability and simplicity, we now approximate pores as straight, tube-like structures. More realistic pore shapes can be investigated analogously using additional geometric correction terms. While the numerical Crank-Nicolson integration routine takes pores of varying diameter into account, for the analytic approach we make additional approximations to increase readability. Since $2\frac{\partial R}{\partial z} \ll 1$, dynamics in pores are faithfully represented by transport dynamics in straight pores of constant radius $\overline{R}$. Furthermore, following our observation that the average pore diameters hardly vary throughout the porous medium, Fig. S3, we consider pores to have the same average radius, $\overline{R}$. This approximation now allows for exact solutions of transport dynamics in temporal Laplace space. Here, transport through the junction is initialised at the entry of the incoming parallel tube

$$s\hat{c}_(x,s) = \delta(x)\delta_{\|,\text{in},1} - \frac{Q_i}{\pi\overline{R}^2}\frac{\partial \hat{c}_i(x,s)}{\partial x} + \kappa\left(1+\frac{Q_i^2}{48\pi^2\overline{R}^2\kappa^2}\right)\frac{\partial^2\hat{c}_i(x,s)}{\partial x^2}, \tag{3}$$

where Q is the flow rate and κ is the molecular diffusibility. Unique solutions follow for absorbing boundary conditions at our two exit locations $\hat{c}_{\|,\text{out}}(\ell_{\|,\text{out}}, t) = \hat{c}_\perp(\ell_\perp, t) = 0$ and an infinite boundary at the inlet accommodating for back-diffusion in the parallel inflow pore. The latter, we achieve by virtually extending the parallel inflow pore to $-\infty$. The algebraic solutions for the Laplace transformed concentrations then determine directly the mean first passage time $\langle t_{\|,\text{out}}\rangle$ and its second moment $\langle t^2_{\|,\text{out}}\rangle$ [5, 2]

$$-\pi\overline{R}^2\kappa\left(1+\frac{Q_i^2}{48\pi^2\overline{R}^2\kappa^2}\right)\frac{\partial\hat{c}_{\|,\text{out}}(x,s)}{\partial x}\Bigg|_{x=\ell/2} = P_{\|,\text{out}}\left(1-\langle t_{\|,\text{out}}\rangle s + \frac{1}{2!}\langle t^2_{\|,\text{out}}\rangle s^2 + ...\right). \tag{4}$$

Results for the mean first passage time $\langle t_\perp\rangle$ and its second moment $\langle t_\perp^2\rangle$ of particles exiting through the perpendicular pore is obtained analogously. These four weighted moments combined give information about mean and

variance of the transit time through a junction block, as will be exemplified in the next section. In particular, the ratio of probabilities to exit through the parallel versus the perpendicular pore is

$$\frac{P_{\|,\mathrm{out}}}{P_{\perp}} = \frac{\left(1 - e^{-(\ell Q \perp)/D_{\perp}}\right) Q_{\|,\mathrm{out}}}{\left(1 - e^{-(\ell Q_{\|,\mathrm{out}})/D_{\|,\mathrm{out}}}\right) Q_{\perp}}, \tag{5}$$

with $D_i = \kappa \left(1 + \frac{Q_i^2}{48\pi^2 \bar{R}^2 \kappa^2}\right)$. This allows using the flow partitioning at a junction to determine which lane of transport dominates. For small and intermediate disorder, we found that flow in parallel pores is much larger than in perpendicular pores $Q_{\|,\mathrm{out}} \gg Q_{\perp}$. Thus, transport is primarily governed by advection in parallel pores. As a consequence, this estimate also asses contributions of the parallel outflow pore to be dominant for both the total mean and variance of the transit time through a pore junction.Importantly, we take from this analysis, that traversing a pore junction can be interpreted as stochastic process. Moreover, we can fully determine the statistical moments of this process, if the flow splitting within the junction is given, i.e. $\{Q_{\|,\mathrm{in}}, Q_{\|,\mathrm{out}}\}$.

1.5 Definition of the total variance of the transit time through a junction unit

In this section, we give a pedagogical approach on how the total variance of the transit time statistics through a junction unit is obtained. For this, we start with a thought experiment: Consider having pore junction blocks all with the same geometry, but differing in their flow splittings. Consider randomly choosing one pore junction unit and letting one solute particle pass through. Repeating this process and noting down transit times, we obtain a distribution from which we estimate a variance. This is the variance of moving forward one building unit. To obtain the variance of passing through the whole porous medium, we need to multiply the variance with the length of the porous medium measured in pore junction building blocks. Here, we made the assumption of large porous media, where particles passing through the entire porous medium effectively sample the whole distribution of flow splittings.

From this thought experiment, we understand that analysing how the total variance of moving forward one building block is sufficient to learn about the efficiency of transport through the full porous medium. Note, however, that the distribution we want obtain by this thought experiment might be complicated in shape and impossible to track analytically. Yet, employing the law of total variance, we can precisely compute the variance of this distribution without the need to fully derive the above mentioned distribution.

While the above mentioned approach is general, now we focus on the specific pore junction geometry consisting of one parallel inflow, one parallel outflow, and one perpendicular pore. Remember, that in each pore junction unit a solute particle can either exit through the outflowing parallel pore or the perpendicular pore. For an illustrative purpose and without loss of generality, we assume that if a particle exiting through the parallel outflow pore leaves the pore junction unit faster than a particle exiting through the perpendicular pore. We assume now in a first step, that all pore junction blocks have the same flow splitting and solute particles take a deterministic time $T_{\{\|,\perp\}}$ to exit through the junction unit the parallel outflow pore or the perpendicular pore. The particle randomly chooses $P_{\{\|,\perp\}}$ to leave through the parallel or the perpendicular pore. The distribution obtained in this case is fully defined by two delta peaks weighted by $P_{\{\|,\perp\}}$. In a next step, we release the assumption of equal junctions and instead consider different flow splittings for different pore junction units. This results in different deterministic transition times $T_{i,\{\|,\perp\}}$ for different pore junctions i. The two delta peaks spread out. Finally, we drop that transition is deterministic, but we have a probability $p(t|\{\|,\perp\},i)$ of passing times through a junction unit, given that we have junction i and exit through the perpendicular or the parallel pore. The distribution we obtain for passing through the pore junction unit is a mixture distribution. To estimate the variance of the mixture distribution, we first estimate the variance of the *two peaks* individually and conclude on the variance of the mixture distribution in a second step. This ultimately justifies why considering exit statistics through the parallel outflow pore can set dominant contributions to the total variance. The overall goal is now to identify how variance and mean of the two peaks vary as function of the pore space heterogeneity. In the following, we describe how one concludes from these quantities on the total variance of the junction unit; Recall, that in the main manuscript we detailed out how the variance of one peak is estimated.

We next turn to derive the variance of the peaks stemming from leaving through the parallel outflow pore or the perpendicular pore. For illustrative purpose, we take a step back and consider deterministic passing times, i.e. $p(t|\{\|,\perp\},i) = \delta(t - T_{i,\{\|,\perp\}})$. For flow splitting statistics summarised by a one-dimensional probability density function, We find for the peaks the following distributions

$$p'_{\{\|,\perp\}}(t) = \int_{-\infty}^{\infty} p(t|\{\|,\perp\},i) P(\{\|,\perp\},i) di \tag{6}$$

$$= \int_{-\infty}^{\infty} \delta(t - T_{i,\{\|,\perp\}}) P(\{\|,\perp\}, i) di \tag{7}$$

$$= \int_{-\infty}^{\infty} \delta(t - T_{i,\{\|,\perp\}}) P(\{\|,\perp\}|i) p(i) di, \tag{8}$$

which describe the already normalised peaks building the mixture distribution. Here, we corrected for the fact, that different junctions have different splitting probabilities $P(\{\|,\perp\}, i)$. For correct normalisation of the peaks to derive the individual probability density functions, we need to correct

$$p_{\{\|,\perp\}}(t) = \frac{p'_{\{\|,\perp\}}(t)}{\int_{-\infty}^{\infty} P(\{\|,\perp\}|i)p(i)di} \tag{9}$$

$$= \frac{p'_{\{\|,\perp\}}(t)}{P(\{\|,\perp\})}, \tag{10}$$

where we defined the weights $P(\{\|,\perp\})$ giving the overall probability to exit through the parallel or the perpendicular pore. Here, $p_{\{\|,\perp\}}(t)$ is the probability distribution underlying a single peak. For mixture distributions consisting of two peaks, we have the following identity valid for any function $H(T)$.

$$E[H(T)] = \int_{-\infty}^{\infty} H(t)(P(\|)p_{\|}(t) + P(\perp)p_{\perp}(t))dt \tag{11}$$

$$= (P(\|)E[H(t_{\|})] + P(\perp)E[H(t_{\perp})]). \tag{12}$$

Here, we defined T as random variable stemming from the mixture distribution, $t_{\{\|,\perp\}}$ as random variable from the peak component distributions. Specifically, for the variance we find

$$\begin{aligned} E[(T-\mu)^2] =& E[T^2] - \mu^2 \\ =& (P(\|)E[(T_{\|})^2] + P(\perp)E[(T_{\perp})^2]) - \mu^2 \\ =& (P(\|)\sigma_{\|}^2 + P(\|)\mu_{\|}^2 + P(\perp)\sigma_{\perp}^2 + P(\perp)\mu_{\perp}^2) - \mu^2 \\ =& (P(\|)\sigma_{\|}^2 + P(\perp)\sigma_{\perp}^2) + P(\|)(\mu_{\|}^2 - \mu^2) + P(\perp)(\mu_{\perp}^2 - \mu^2) \end{aligned} \tag{13}$$

If there is an additional variance on $t_{\{\|,\perp\}}$, $p'_{\{\|,\perp\}}(t)$ changes, while the subsequent calculations remain valid. Note, that we only need to compute $\mu_{\{\|,\perp\}}$ and $\sigma_{\{\|,\perp\}}$, and not the full probability $p'_{\{\|,\perp\}}(t)$. Specifically, $\sigma_{\{\|,\perp\}}$ is the total variance. Note, that we can directly estimate $\mu'_{\{\|,\perp\}}$ and $\sigma'_{\{\|,\perp\}}$ as corrections for the division and subsequent multiplication with the weights $P(\{\|,\perp\})$ cancel. In the main text, we explained how estimates for $\mu'_{\{\|,\perp\}}$ and $\sigma'_{\{\|,\perp\}}$ are derived.

Specifically for porous media of low and medium disorder, the series expansion over $\mathrm{Var}[T_{\|,\mathrm{out}}]$ needs to take care of the weights arising from flow splittings. Explicitly, $\mathrm{Var}^{\mathbf{P}}[T_{\|,\mathrm{out}}|\{Q_{\|,\mathrm{in}}, Q_{\|,\mathrm{out}}\}]$ is given by

$$\mathrm{Var}^{\mathbf{P}}[T_{\|,\mathrm{out}}|\{Q_{\|,\mathrm{in}}, Q_{\|,\mathrm{out}}\}] = \left.\frac{\partial^2 f_{\|}(s)}{\partial s^2}\right|_{s=0} - \frac{1}{f_{\|}(0)}\left(\left.\frac{\partial f_{\|}(s)}{\partial s}\right|_{s=0}\right)^2, \tag{14}$$

$$\mathrm{E}^{\mathbf{P}}[T_{\|,\mathrm{out}}|\{Q_{\|,\mathrm{in}}, Q_{\|,\mathrm{out}}\} = -\left.\frac{\partial f_{\|}(s)}{\partial s}\right|_{s=0}, \tag{15}$$

$$\mathrm{Var}^{\mathbf{P}}[T_{\perp,\mathrm{out}}|\{Q_{\|,\mathrm{in}}, Q_{\|,\mathrm{out}}\}] = \left.\frac{\partial^2 f_{\perp}(s)}{\partial s^2}\right|_{s=0} - \frac{1}{f_{\perp}(0)}\left(\left.\frac{\partial f_{\perp}(s)}{\partial s}\right|_{s=0}\right)^2 + \Gamma_{\sigma^2}, \tag{16}$$

$$\mathrm{E}^{\mathbf{P}}[T_{\perp,\mathrm{out}}|\{Q_{\|,\mathrm{in}}, Q_{\|,\mathrm{out}}\}] = -\left.\frac{\partial f_{\perp}(s)}{\partial s}\right|_{s=0} + \Gamma_{\mu}, \tag{17}$$

$$\tag{18}$$

where we used $f_{\|}(s) = -A_{\|,\mathrm{out}} D_{\|,\mathrm{out}} \left.\frac{\partial \hat{c}_{\|,\mathrm{out}}(x,s)}{\partial x}\right|_{x=\ell/2}$ and $f_{\perp}(s) = -A_{\perp} D_{\perp} \left.\frac{\partial \hat{c}_{\perp}(x,s)}{\partial x}\right|_{x=\ell}$ for abbreviation. Note, that we added Γ_{σ^2} and Γ_{μ} to correct for the chosen junction geometry. These two contributions correct for the fact that after leaving the perpendicular pore, the particle still needs to travel half the parallel inflow pore up to reach the same starting point for the next pore juncton unit block. We estimate these contributions to

$$\Gamma_\mu = P(\perp) \cdot \frac{\bar{l}}{2\overline{v_\|}} \tag{19}$$

$$\Gamma_{\sigma^2} = P(\perp) \cdot \frac{\overline{D_{\mathrm{eff}}}\bar{l}}{\overline{v_\|}^3}. \tag{20}$$

Here $\overline{v_\|}$ denotes the average flow velocity over all parallel pores. $\overline{D_{\mathrm{eff}}}$ is computed using the averages over all parallel pores.

1.6 Expansion of the pore junction transition time moments

We obtain $\mathrm{E}^{\mathrm{p}}[T_{\|,\mathrm{out}}|\{Q_{\|,\mathrm{in}}, Q_{\|,\mathrm{out}}\}]$ and $\mathrm{Var}^{\mathrm{p}}[T_{\|,\mathrm{out}}|\{Q_{\|,\mathrm{in}}, Q_{\|,\mathrm{out}}\}]$ from the first passage time formalism. Here, we understand the mean and the variance as function of the flow splitting $\{Q_{\|,\mathrm{in}}, Q_{\|,\mathrm{out}}\}$. Taking the flow splitting as random variable, we can expand both the mean and the variance around these two random variables. Specifically, we take $\{Q_{\|,\mathrm{in}}, Q_{\|,\mathrm{out}}\}$ as a multivariate Gaussian centered at the average flow (Q_0, Q_0). Defining $q_1 = (Q_{\|,\mathrm{in}} + Q_{\|,\mathrm{out}})/2Q_0$ and $q_2 = (Q_{\|,\mathrm{in}} - Q_{\|,\mathrm{out}})/2Q_0$ as independent variables associated with the two eigenvalues $\sigma_1 = \alpha_1\chi$, $\sigma_2 = \alpha_2\chi$. Up to fourth order we obtain for the mean of the variance

$$\begin{aligned}
&\mathrm{E}^{\mathrm{j}}[\mathrm{Var}^{\mathrm{p}}[T_{\|,\mathrm{out}}|\{Q_{\|,\mathrm{in}}, Q_{\|,\mathrm{out}}\}]] = \\
&\quad \mathrm{V}(Q_0, Q_0) \\
&\quad + \frac{1}{2}\left(\alpha_1^2 \mathrm{V}_{11}(Q_0, Q_0) + \alpha_2^2 \mathrm{V}_{22}(Q_0, Q_0)\right)\chi^2 \\
&\quad + \frac{1}{24}\left(\alpha_1^4 \mathrm{V}_{1111}(Q_0, Q_0) + 4\alpha_2^2\alpha_1^2 \mathrm{V}_{1122}(Q_0, Q_0)\right. \\
&\qquad \left. + \alpha_2^4 \mathrm{V}_{2222}(Q_0, Q_0)\right)\chi^4 \\
&\quad + \mathcal{O}(\chi^6),
\end{aligned} \tag{21}$$

where $\mathrm{V}(Q_{\|,\mathrm{in}}, Q_{\|,\mathrm{out}}) = \mathrm{Var}^{\mathrm{p}}[T_{\|,\mathrm{out}}|\{Q_{\|,\mathrm{in}}, Q_{\|,\mathrm{out}}\}]$ was used for readability. Subscripts indicate derivatives with respect to q_1, q_2. Note, that terms of odd order vanish for expansions of Gaussian random variables. Here, we define the three coefficients $\Lambda_0^{\mathrm{V}_\|}, \Lambda_2^{\mathrm{V}_\|}$, and $\Lambda_4^{\mathrm{V}_\|}$ summarising the coefficents for the respective order in χ^k.

Next, we estimate theses coefficients for large Peclet numbers, $\mathrm{Pe} \gg 1$. To this end, we compare with Fig. S4 b, d, f. We identify that the contribution of $\Lambda_0^{\mathrm{V}_\|}$ is non-vanishing and positive, $\Lambda_2^{\mathrm{V}_\|}$ gives a strong negative contribution, and that higher derivatives are vanishing and hence $\Lambda_4^{\mathrm{V}_\|}$ is vanishing. Specifically, we can trace back the negative contribution to originate from altering q_2, which in turn is connected to the splitting of the flow between the parallel and the perpendicular outflow pore. Importantly, the variance of exiting though the parallel outflow pore has a maximum for $q_2 = 0$, which comes with a simple physical interpretation. Though transport by advection through the parallel pores being the dominant transport mode, the variance of this movement is strongly influenced by particles getting *trapped* in perpendicular pores. Changing the flow in the perpendicular pores has a strong effect on the efficiency of transport, as the trapping mechanisms are altered: Outflow perpendicular pores reduce back diffusion and trapped particles are transported through the pore to the next junction, while inflow perpendicular pores reduce the probability to be trapped in the perpendicular pore in the first place. These effects are not captured if transit time statistics of pores instead of pore junctions are considered.

We perform an analogous analysis for $\mathrm{Var}^{\mathrm{j}}[\mathrm{E}^{\mathrm{p}}[T_{\|,\mathrm{out}}|\{Q_{\|,\mathrm{in}}, Q_{\|,\mathrm{out}}\}]]$, and obtain

$$\begin{aligned}
&\mathrm{Var}^{\mathrm{j}}[\mathrm{E}^{\mathrm{p}}[T_{\|,\mathrm{out}}|\{Q_{\|,\mathrm{in}}, Q_{\|,\mathrm{out}}\}]] = \\
&\quad (\alpha_1^2 {\mathrm{E}_1}^2(Q_0, Q_0) + \alpha_2^2 {\mathrm{E}_2}^2(Q_0, Q_0))\chi^2 \\
&\quad + \left(\frac{\alpha_1^4}{2}\left({\mathrm{E}_{11}}^2(Q_0, Q_0) + \frac{2}{3}\mathrm{E}_{1111}(Q_0, Q_0)\right)\right. \\
&\qquad + \alpha_1^2\alpha_2^2 \mathrm{E}_{122}(Q_0, Q_0)\mathrm{E}_1(Q_0, Q_0) \\
&\qquad + \alpha_1^2\alpha_2^2 {\mathrm{E}_{12}}^2(Q_0, Q_0) \\
&\qquad + \alpha_1^2\alpha_2^2 \mathrm{E}_{112}(Q_0, Q_0)\mathrm{E}_2(Q_0, Q_0) \\
&\qquad \left. + \frac{\alpha_2^4}{2}\left({\mathrm{E}_{22}}^2(Q_0, Q_0) + \frac{2}{3}\mathrm{E}_{2222}(Q_0, Q_0)\right)\right)\chi^4 + \mathcal{O}(\chi^6),
\end{aligned} \tag{22}$$

where $\mathrm{E}(Q_{\parallel,\mathrm{in}}, Q_{\parallel,\mathrm{out}}) = \mathrm{E}^{\mathrm{p}}[T_{\parallel,\mathrm{out}}|\{Q_{\parallel,\mathrm{in}}, Q_{\parallel,\mathrm{out}}\}]$ was used for readability. We define the two coefficients $\Lambda_2^{\mathrm{E}_\parallel}$, and $\Lambda_4^{\mathrm{E}_\parallel}$. Also estimating the contributions of the coefficients for large Peclet numbers Pe $\gg 1$, we compare with Fig. S4 a, c, e (blue line). Here, we find that the first order derivatives are small, which yields $\Lambda_2^{\mathrm{E}_\parallel}$ to be a small positive contribution. In contrast, for $\Lambda_4^{\mathrm{E}_\parallel}$ the square of the second order derivative with respect to q_2 dominates. This renders $\Lambda_4^{\mathrm{E}_\parallel}$ a strong positive contributions. We find that the transport efficiency predominantly decreases due to the variance between different parallel pathways through the porous medium.

The discussion for the perpendicular outflow pores is analogous, also visible in Fig. S4 c, d, e, f (green line). While contributions from the perpendicular outflow add correction terms, the qualitative behaviour remains unchanged. Having identified the dominate contributions that give rise to the non-monotonous dependency between transport efficiency and flow heterogeneity for large Peclet numbers Pe $\gg 1$, we ask how these contributions change as the Peclet number is reduced to low Peclet numbers Pe $\ll 1$. Putting a special focus on the second derivative of the variance of the pore junction passage time with respect to q_2, we find that the contribution changes signs for low Pe $\ll 1$, as demonstrated in Fig. S5. To this end, the relative change of the flow direction averaged variance

$$\mathrm{Var}^{\mathrm{p},*}[T_{\parallel,\mathrm{out}}|\{q_1, q_2\}] = \frac{\mathrm{Var}^{\mathrm{p}}[T_{\parallel,\mathrm{out}}|\{q_1, q_2\}] + \mathrm{Var}^{\mathrm{p}}[T_{\parallel,\mathrm{out}}|\{q_1, -q_2\}]}{2} \tag{23}$$

is inspected for its relative change as q_2 is increased. For visualisation purpose, the monotonic function $m[x] = \exp\left((x - x_0)/x_0\right) - 1$ is used to depict whether $\mathrm{Var}^{\mathrm{p},*}[T_{\parallel,\mathrm{out}}|\{q_1, q_2\}]$ has a positive or negative second derivative with respect to q_2. The dotted line in Fig.S5 c demonstrates that the second order derivative is only negative above a critical Pe_c, such that we only expect a non-monotonic dependency between transport efficiency and flow heterogeneity for large Peclet numbers.

1.7 Fitting of simulation data and semi-numeric approximation of non-monotonicity

In numerical simulations and experiments, we determined the front width as direct deviation from a step function profile. For the analytic approach, we presented an expansion of the variance of the passage time as an even function of fourth order. As exemplified in section 1.8, we draw a proportionality between the front width and the standard deviation of the passage time statistics. Now, to convert the variance to the standard deviation, the root of the expansion needs to be considered. Importantly, taking the root of an even expansion that is restricted to $\mathbb{R}^+$ both in the variable and the function space leaves the functional form unaffected:

$$\sqrt{a - bx^2 + cx^4 + dx^6} = \sqrt{a} - \frac{b}{2\sqrt{a}}x^2 + \frac{(ac - b^2)}{8\sqrt{a^3}}x^4 + O(x^6). \tag{24}$$

We find that the functional form of the expansion still predicts the qualitative shape of the function, which justifies the fit presented in Fig. 1 c in the main manuscript.

To compare the predicted shape of the non-monotonocity by expansion of the total variance and the simulation fit, we focus on the squared front width w^2 to allow for a direct proportionality with the total variance. Fitting a function of the form $f(x) = a + bx^2 + cx^4$ to these transformed variables in the range $q \in [0, 0.3]$, we find $a = 774\,\mathrm{\mu m}^2$, $b = -3005\,\mathrm{\mu m}^2$, and $c = 18\,222\,\mathrm{\mu m}^2$ with an adjusted $R^2 = 96\%$. This results in a non-monotonicity with a minimum at $\chi_{\min} \approx 0.15$. The decrease from $\chi = 0$ to $\chi_{\min}$ is $f(\chi_{\min})/f(\chi = 0) = 84\%$.

Using the moments of the transit time through pore junction building blocks, we get estimates for the quartic expansion and compare them with the fit to the data. As a full functional computation of the moments is infeasible, we rely on a semi-numeric approach. As first approximation, we recognise that the major contribution to the total variance is stemming from contributions of varying the flow along the direction of q_2. Making use of the proportionality between disorder and the flow heterogeneity for low disorder, we convert deviations in the flow disorder σ_q/Q_0 to disorder χ, as already introduced in the main text. We take exiting through the parallel tube, exiting through the perpendicular tube, and the difference in mean transit time of exiting through the parallel versus the perpendicular outflow pore $P(\parallel)(\mu_\parallel^2 - \mu^2) + P(\perp)(\mu_\perp^2 - \mu^2)$ into account. Here, we treat these three respective contributions separately.

To generate semi-numeric results, we fix the tube length l, tube radius r, the mean parallel flow velocity u, and the molecular diffusivity k to constant values yield a Péclet number $Pe_\parallel = lu/k = 30$. Specifically, we choose the unitless values $l = 2.4$, $r = 2.0$, $u = 0.5$, and $k = 0.04$ granting numerical stability. We checked, that changes in parameters only have a small effect on the final result, as long as the Péclet number is fixed. For fixed values, the functions $\mathrm{E}^{\mathbf{P}}[T_{(i)}|\{Q_0, q_2\}]$ and $\mathrm{Var}^{\mathbf{P}}[T_{(i)}|\{Q_0, q_2\}]$ are evaluated numerically. To compute the contributions for

Table S1: **Quartic fit on semi-numeric integration** The quartic fits are of the functional form $f(x) = a + bx^2 + cx^4$. All parameter here are in the units of μm^2 obtained by fitting the offset to the fit to the numerical experimental data. Here, $\delta M[q_2]$ describes how the difference in mean transit time of exiting through the parallel versus the perpendicular outflow pore $P(\|)(\mu_\|^2 - \mu^2) + P(\perp)(\mu_\perp^2 - \mu^2)$ changes with varying disorder taking only contributions from varying q_2 into account.

	a	b	c
$\mathrm{E^f}[\mathrm{Var^P}[T_{\|,\mathrm{out}}\|\{Q_0, q_2\}]]$	500	−2580	14800
$\mathrm{Var^f}[\mathrm{E^P}[T_{\|,\mathrm{out}}\|\{Q_0, q_2\}]]$	0	120	250
$\mathrm{E^f}[\mathrm{Var^P}[T_{\perp,\mathrm{out}}\|\{Q_0, q_2\}]]$	85	−130	1635
$\mathrm{Var^f}[\mathrm{E^P}[T_{\perp,\mathrm{out}}\|\{Q_0, q_2\}]]$	0	380	30
$\delta M[q_2]$	190	−1990	11860

the variance, we multiply the numerical functions with the probability density function of Gaussian distributions $\mathcal{N}(0, (q_2/Q_0)^2)$ and integrate the result. According to the expansion, we fit the approximations with even quartic polynomials. The quality of this fitting procedure is exemplified in Fig. S5 a. The results of the fits are summarised in table S1. From this, we correctly predict the order of magnitude of the initial monotonicity with a dip of $\chi^{\mathrm{t}}_{\min}/\chi^{\mathrm{t}}_0 \approx 80\%$ at a disorder of $\chi^{\mathrm{t}}_{\min} = 19.5\%$ in agreement with the fit to the data. Note, that additional contributions from q_1 and cross-terms consisting of derivatives of q_1 and q_2 are shifting to smaller dips at smaller disorder values.

1.8 Connetcion between pdf, cdf, and fpt in 1D

Here, we illustrate the connection between occupation probability, survival probability, and first passage time in a one-dimensional system and by this establish a connection between the experimental, simulation, and analytic measure. We want to emphasise, that the approach presented here focuses on the variance, but can be easily extended to moments of higher order.

We place a diffusive particles at $x_0 < 0$ and implement an absorbing boundary condition at $x_c = 0$. We allow diffusion of particles to $-\infty$, such that we consider the motion of the particles in the interval $(-\infty, 0]$. We set a drift that drives the particles to the absorbing boundary $v > 0$. The probability to find the diffusive particle at position x at time t is given by

$$\frac{\partial p(x,t)}{\partial t} = -v\frac{\partial p(x,t)}{\partial x} + D\frac{\partial^2 p(x,t)}{\partial x^2}, \tag{25}$$

where D is the diffusion coefficient of the particle. For the absorbing boundary condition we have the condition $p(0,t) = 0 \quad \forall \quad t > 0$. The initial condition is given by $p(x,0) = \delta(x_0 - x) \quad \forall \quad x < 0$. This equation is solved using the method of images

$$\begin{aligned} p(x,t) =& \frac{1}{\sqrt{4\pi Dt}}\left(\exp\left(-\frac{(x - x_0 - vt)^2}{4Dt}\right)\right. \\ &\left. - \exp\left(-\frac{4x_0 v}{4D}\right)\exp\left(-\frac{(x + x_0 - vt)^2}{4Dt}\right)\right). \end{aligned} \tag{26}$$

We define the Peclet number $\mathrm{Pe} = x_0 v/D$. A measure for the deviation of the mean motion of the tracer particle is computing the variance of the this probability density. For motion far away from the absorbing boundary, i.e. $vt \ll -x_0$ and $Pe \gg 1$, the second term in the probability density is only a small correction. Under these assumptions, the variance is approximated by $\sigma_x \approx \sqrt{2Dt}$.

The cumulative distribution function $S(x,t)$ is defined as

$$S(x,t) = \int_{-\infty}^{x} \mathrm{d}x' p(x',t), \tag{27}$$

which results for $p(x,t)$ defined above in

$$S(x,t) = \frac{1}{2}\left(\mathrm{erfc}\left[\frac{tv + x_0 - x}{\sqrt{4Dt}}\right] - \exp\left(-\frac{vx_0}{D}\right)\mathrm{erfc}\left[\frac{tv - x_0 - x}{\sqrt{4Dt}}\right]\right), \tag{28}$$

where we made use of the complementary error function. The survival probability is giving the probability that the diffusive particle is still in the interval $(-\infty, 0]$ at time t. The survival probability is a special case of the cumulative distribution function $S(t) = S(0, t)$ evaluated at the absorbing boundary. The cumulative distribution function thus gives the same information as the occupation probability $p(x, t)$. While for the occupation probability a delta peak disperses over time, for the cumulative distribution function a step function "smears out" over time. As for the occupation probability, we define a measure for deviation of the mean in the cumulative distribution function. Here, we measure the width of the dispersive step, e.g. we measure the interval between $S(x, t) = 0.25$ and $S(x, t) = 0.75$. Evaluating as before for $Pe \gg 1$ and $vt \ll -x_0$ we find $\Delta x \approx 4\sqrt{Dt} \cdot \mathrm{erfc}^{-1}(0.5)$. Note, that σ_x and Δx have the same scaling with respect to the diffusion coefficient D.

The first passage probability $f(t)$ gives the probability that the diffusive particles reaches the absorbing boundary for the first time at time t. By evaluating the temporal change of survival probability, the first passage probability is defined as

$$f(t) = -\frac{\partial S(t)}{\partial t}. \tag{29}$$

For the above defined setup, we find

$$f(t) = -\frac{Dx_0}{\sqrt{4\pi D^3 t^3}} \exp\left(\frac{(vt + x_0)^2}{4Dt}\right). \tag{30}$$

The first passage time is distributed like an inverse gaussian variable $T \sim IG(x_0/v, x_0^2/(2D))$. A measure for the deviation of the mean is obtained from the variance of this distribution which scales like $\sigma_t = \sqrt{2Dx_0/v^3}$. We recover once again the same scaling with resepect to the diffusion coefficient D as for σ_x and Δx. For a one dimensional system, we obtain estimates an effective diffusion coefficient D thus from the first passage time, the cumulative distribution function, or the occupation probability. A larger variance in the first passage time thus implies an increased effective diffusion and likewise an increased width of the dispersive front in the picture of cumulative distribution function.

In the work here, we employ this analogy to gain mechanistic understand of how effective diffusion coefficients of the porous medium scale with the microscopic pore statistics. Employing the law of large numbers to more complex settings justifies an effective mapping to Gaussian statistics to establish a connection between the standard deviation in the transit time statistics and an effective front width for large porous media. Importantly, we only give information about the qualitative scaling and make no direct conversion between σ_t and Δ_x. Understanding that changing the disorder has effect on a effective diffusion through the porous medium on the macroscopic medium scale, we translate the functional dependence between σ_t to an effective diffusion and to Δx, as σ_t and Δx have the same scaling with respect to an effective diffusion.

1.9 Splitting the movement into sub-steps

Here, we motivate the boundary condition allowing diffusion back to $-\infty$. For one-dimensional settings, this boundary condition allows to split the motion from x_0 to x_c into an arbitrary number of steps. To exemplify this, consider splitting the interval into an arbitrary number of sub-steps $x_0 < x_1 < x_2 < ... < x_n < x_{n+1} = x_c$. In first step, we let the particle start at x_0 and with an absorbing boundary at x_1, in a second step we let the particle start at x_1 and we have an absorbing boundary at x_2, ... We easily see that the distance the particle needs to travel per step is given by

$$\delta x_i = x_{i+1} - x_i = \frac{x_{i+1} - x_i}{x_c - x_0}(x_c - x_0) = w_i \delta x_c,$$
$$\sum_i^n w_i = 1, \tag{31}$$

and the total time to traverse the interval $[x_0, x_c]$ is given by $T = \sum T_i$, where $T_i \sim IG(w_i \delta x_c/v, w_i^2 \delta x_c^2/(2D))$ are distributed as inverse Gaussian distributions, if for each step back diffusion to $-\infty$ is allowed. Making use of the properties of the inverse Gaussian distribution, we find $T \sim IG(\sum w_i \delta x_c/v, (\sum w_i)^2 \delta x_c^2/(2D))$. Using the fact, that the sum $\sum w_i$ by construction equals to unity, the result simplifies to $T \sim IG(\delta x_c/v, \delta x_c^2/(2D))$. Hence, no matter how many sub-steps we consider, the results is the same as if the interval is considered in one single step. Note, that argument doesn't hold, if a reflecting boundary condition at x_0 is applied.

Supplementary References

[1] K. Alim, S. Parsa, D. A. Weitz, and M. P. Brenner. Local Pore Size Correlations Determine Flow Distributions in Porous Media. *Phys. Rev. Lett.*, 119(14):144501, Oct. 2017. Publisher: American Physical Society.

[2] L. de Arcangelis, J. Koplik, S. Redner, and D. Wilkinson. Hydrodynamic Dispersion in Network Models of Porous Media. *Phys. Rev. Lett.*, 57(8):996–999, Aug. 1986. Publisher: American Physical Society.

[3] S. Marbach and K. Alim. Active control of dispersion within a channel with flow and pulsating walls. *Phys. Rev. Fluids*, 4(11):114202, Nov. 2019. Publisher: American Physical Society.

[4] G. N. Mercer and A. J. Roberts. A Centre Manifold Description of Contaminant Dispersion in Channels with Varying Flow Properties. *SIAM J. Appl. Math.*, 50(6):1547–1565, Dec. 1990. ISSN 0036-1399. Publisher: Society for Industrial and Applied Mathematics.

[5] S. Redner. *A Guide to First-Passage Processes.* Cambridge University Press, Cambridge, 2001. ISBN 978-0-521-65248-3.

[6] Q. Xiong, T. G. Baychev, and A. P. Jivkov. Review of pore network modelling of porous media: Experimental characterisations, network constructions and applications to reactive transport. *Journal of Contaminant Hydrology*, 192:101–117, Sept. 2016. ISSN 0169-7722.

[7] A. Zareei, D. Pan, and A. Amir. Temporal Evolution of Flow in Pore-Networks: From Homogenization to Instability. *arXiv:2106.09745 [cond-mat, physics:physics]*, June 2021. arXiv: 2106.09745.
